# *MiR-584-5p* potentiates vincristine and radiation response by inducing spindle defects and DNA damage in medulloblastoma

**DOI:** 10.1038/s41467-018-06808-8

**Published:** 2018-10-31

**Authors:** Nourhan Abdelfattah, Subapriya Rajamanickam, Subbarayalu Panneerdoss, Santosh Timilsina, Pooja Yadav, Benjamin C. Onyeagucha, Michael Garcia, Ratna Vadlamudi, Yidong Chen, Andrew Brenner, Peter Houghton, Manjeet K. Rao

**Affiliations:** 1 Greehey Children’s Cancer Research Institute, University of Texas Health Science Center at San Antonio, San Antonio, TX 78229 USA; 2 Department of Cell Systems and Anatomy, University of Texas Health Science Center at San Antonio, San Antonio, TX 78229 USA; 3 Department of Chemistry, Faculty of Science, Cairo University, Cairo, 12613 Egypt; 4 Department of Molecular Medicine, University of Texas Health Science Center at San Antonio, San Antonio, TX 78229 USA; 5 Department of Medicine, University of Texas Health Science Center at San Antonio, San Antonio, TX 78229 USA; 6 Department of Obstetrics and Gynecology, University of Texas Health Science Center at San Antonio, San Antonio, TX 78229 USA; 7 Department of Epidemiology and Statistics, University of Texas Health Science Center at San Antonio, San Antonio, TX 78229 USA

## Abstract

Despite improvements in overall survival, only a modest percentage of patients survives high-risk medulloblastoma. The devastating side effects of radiation and chemotherapy substantially reduce quality of life for surviving patients. Here, using genomic screens, we identified *miR-584-5p* as a potent therapeutic adjuvant that potentiates medulloblastoma to radiation and vincristine. *MiR-584-5p* inhibited medulloblastoma growth and prolonged survival of mice in pre-clinical tumor models. *MiR-584-5p* overexpression caused cell cycle arrest, DNA damage, and spindle defects in medulloblastoma cells. *MiR-584-5p* mediated its tumor suppressor and therapy-sensitizing effects by targeting HDAC1 and eIF4E3. *MiR-584-5p* overexpression or HDAC1/eIF4E3 silencing inhibited medulloblastoma stem cell self-renewal without affecting neural stem cell growth. In medulloblastoma patients, reduced expression of *miR-584-5p* correlated with increased levels of HDAC1/eIF4E3. These findings identify a previously undefined role for *miR-584-5p*/HDAC1/eIF4E3 in regulating DNA repair, microtubule dynamics, and stemness in medulloblastoma and set the stage for a new way to treat medulloblastoma using *miR-584-5p*.

## Abstract (web summary)

The radiation and chemotherapy used for treating medulloblastoma patients cause debilitating side effects. Here, the authors show that miR-584 acts as a therapeutic adjuvant as it sensitizes medulloblastoma to radiation and chemotherapy by targeting HDAC1 or eIF4E3 to enhance spindle defects and DNA damage.

## Introduction

Medulloblastoma (MB) is the most common malignant pediatric brain cancer, which accounts for >10% of childhood cancer death^1,2^. MB is classified into four molecular sub-types: WNT, Sonic Hedgehog (SHH), group 3, and group 4^3,4^. Group 3 has the worst prognostic outcome, with 40–45% of patients presenting with metastatic lesions^5^. MB is deemed high risk when the tumor is metastatic, has large cell/anaplastic phenotype, or is c-Myc amplified^6^. Despite the progress made in treating MB, the 5-year survival rate for high-risk tumors remains poor and risk of recurrence within 2 years of treatment is still high^7^. Because of the highly toxic side effects of radiation and chemotherapy, surviving children have reduced quality of life. For example, craniospinal exposure of 35.5 Gy followed by up to 54-Gy radiation boosts to the posterior fossa leads to severe neurocognitive deficits, chronic neuropathy, and chronic hypopituitarism as well as secondary malignancies^8^. Similarly, vincristine (VCR)—a microtubule-interfering chemotherapeutic agent routinely administered as a radiotherapy adjuvant to both high-risk and average-risk MB patients^9,10^—causes cumulative dose-dependent neurotoxicity that includes, but is not limited to, sensorimotor and autonomic neuropathy, hearing loss, mononeuropathy, and seizures^11^. Those facts underline the importance of identifying new targets that can serve as more effective and less toxic therapeutics to treat MB.

MicroRNAs (miRNAs) represent one such possibility because they play key roles in chemosensitivity and radiosensitivity. In this study, using a high-throughput miRNA mimic screen, we identified *miR-584-5p* as a potent tumor suppressor that makes VCR and ionizing radiation (IR) more effective in treating MB. Although *miR-584-5p* acts as a tumor suppressor in renal cell carcinoma, glioma, and neuroblastoma^12–14^, no one to our knowledge has investigated its role as a therapeutic adjuvant and underlying mechanism of action in cancer in general and MB in particular. We show that *miR-584-5p* mediates its tumor suppressor and VCR/IR-potentiating effect by targeting eukaryotic translation initiation factor 4e family member 3 (eIF4E3) and histone deacetylase 1 (HDAC1), thereby affecting cell cycle progression, microtubule dynamics, and DNA damage response. Our study reveals that HDAC1 promotes MB growth. Previous studies have shown that eIF4E3 is a translation initiation protein that may act as a tumor suppressor^15,16^. Our study shows a tumor-promoting and chemotherapy/IR-potentiating functions for eIF4E3 in MB. Furthermore, our study is significant as it shows that a tumor suppressor miRNA can sensitize both VCR and IR response by inducing spindle defects and mitotic catastrophe as well as DNA damage in MB.

## Results

### Identification of *miR-584-5p* as a new therapeutic adjuvant

**Fig. 1:**
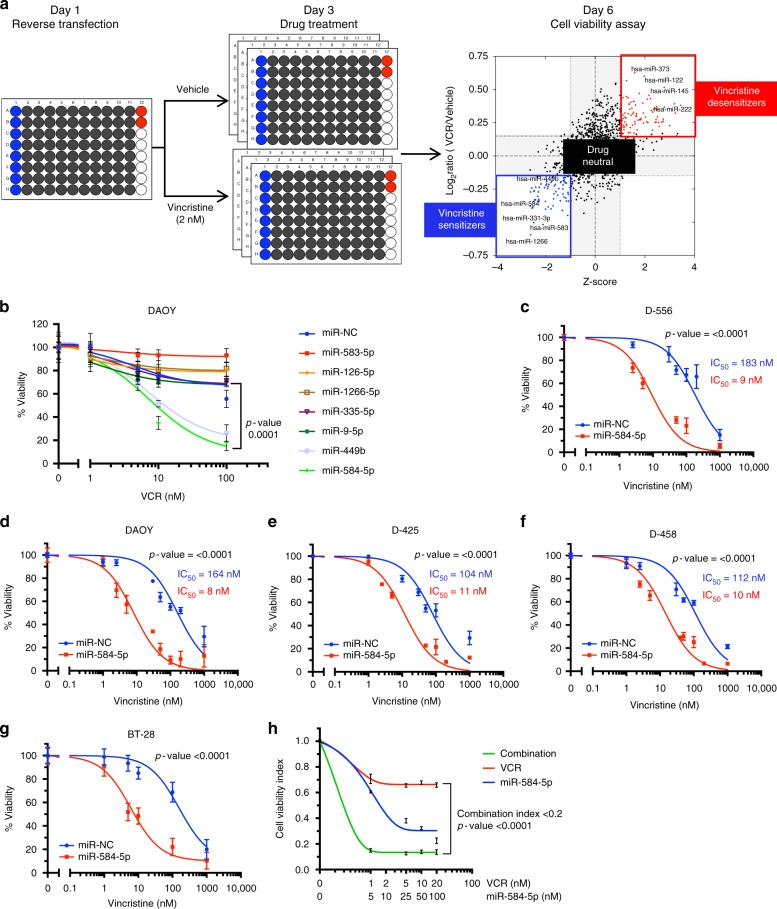
Functional screen identifies *miR-584-5p* as a new therapeutic adjuvant in MB. **a** Outline of the primary screen and list of drug-sensitizer, drug-desensitizer, and drug-neutral miRNAs. A total of 1902 miRNA mimics arrayed in 96-well plates were screened in triplicates. **b** Line graphs showing relative viability of DAOY cells transfected with miR-NC or indicated VCR-sensitizer miRNAs (*miR-584-5p*, *miR-1266, miR-126-5p, miR-335-5p, miR-9-5p, miR-449b*; obtained from primary screen) for 24 h followed by treatment with vehicle or increasing concentration of VCR for 72 h. Cell viability was assessed using alamarBlue® cell viability assay. *p*-value was calculated using one-way ANOVA followed by Dunnett’s multiple-comparisons test. Error bars represent the mean ± SEM of three independent experiments (performed in sixtuplicate for each experiment). **c**–**g** Dose–response curves showing VCR sensitivity in miRNA negative control (NC) (blue line) and *miR-584-5p* mimic-transfected D556Med, D458Med, D425Med, DAOY, and primary MB BT-28 cells. MB cells were transfected with miR-NC or miR-584 mimic followed by treatment with VCR or vehicle for 72 h. Cell viability was assessed using alamarBlue cell viability assay. The *p*-values were determined by the sum-of-squares *F* test. Error bars represent mean ± standard error of the mean (SEM) of three independent experiments (performed in sixtuplicate for each experiment). **h** Synergistic effect of *miR-584-5p* with VCR. D556Med cells were treated with increasing concentrations of *miR-584-5p* and VCR before being subjected to cell viability assay using alamarBlue cell viability assay. Compusyn software (http://www.combosyn.com/) was used to calculate combination indices (CIs). The *p*-values were determined by the sum-of-squares *F* test. Error bars represent mean ± SEM of three independent experiments (performed in sixtuplicate for each experiment)

To identify miRNAs that may sensitize VCR response in MB, we combined a high-throughput screening platform with a library of 1902 chemically synthesized human miRNA mimics (Fig. 1a and Supplementary Fig. 1a–d). The miRNAs are arrayed in a one-miRNA–one-well format in 96-well microtiter plates. Reverse transfection of Group 3/c-Myc-amplified D458Med cells was performed in triplicate in the presence and absence of a sub-lethal concentration of VCR, which was optimized in four MB cell lines before the screen (Fig. 1a and Supplementary Fig. 1b). Cells were subjected to VCR at an IC20 lethal concentration for 72 h after 48 h of transfection, and cell viability was measured (Fig. 1a). Candidate miRNAs were prioritized for validation by functional and interaction assays using standard Student *t*-tests (with pooled variance), false discovery rate (*Q* = 0.5%), and by using the magnitude of response (lowest 2.5 percentile of the distribution of the *μ*_VCR_/*μ*_Control_ ratios).

Our screen yielded three categories of miRNAs:

1. Sensitizers, which decreased the MB cell viability in the presence of VCR in comparison with vehicle;
2. Desensitizers, which increased MB cell viability in the presence of VCR compared in comparison with vehicle; and
3. Drug neutral, which either significantly (>25%) increased or decreased cell viability in vehicle itself and therefore did not affect VCR therapy (Fig. 1a and Supplementary Fig. 1a).

We focused on drug-sensitizer miRNAs that showed significant difference in cell viability in VCR-treated MB cells in comparison with vehicle-treated cells in our primary screen. In our secondary screen, of all the top hits of drug-sensitizer miRNAs tested, miR-584-5p showed the most consistent and comparatively higher magnitude of difference in cell viability in VCR-treated MB cells (Fig. 1b and Supplementary Fig. 1e–f). To further confirm the VCR-sensitizing effect of *miR-584-5p*, we treated MB cells (D425Med, D556Med, D458Med, and DAOY) with *miR-584-5p* mimic in the absence and presence of increasing concentrations of VCR. Combining *miR-584-5p* and VCR resulted in ~10-fold to 20-fold lower 50% inhibitory concentration than that in vehicle in all four cell lines (Fig. 1c–f). Next, we tested the VCR-sensitizing effects of miR-584-5p on primary MB cells isolated from medulloblastoma patient-derived PDXs (BT-28 and BT-50). BT-28 cells are derived from medulloblastoma patient with nodal dysmoplasia and >200 deleterious mutations including mutS homolog 6 (MSH6) and mutL homolog 1 (MLH1); while BT-50 is derived from SHH medulloblastoma with mutations in genes including suppressor of fused homolog (SUFU) and F-Box and WD repeat containing 7 (FBXW7). Similar to MB cell lines, miR-584-5p significantly inhibited the viability of BT-28 and BT-50 MB cells (Fig. 1g and Supplementary Fig. 1g). In addition to inhibiting growth, miR-584-5p potentiated the VCR response in BT-50 and BT-28 (Fig. 1g and Supplementary Fig. 1g). Consistent with that finding, combination treatment with increasing concentration of *miR-584-5p* mimic and increasing concentrations of VCR showed a highly synergistic effects on MB cells for all treatment combinations (www.combosyn.com)^17^ (Fig. 1h, and Supplementary Fig. 1h–l). We wondered whether anti-growth and VCR-sensitizing effects of *miR-584-5p* may also be relevant in other cancers. Our results revealed that like medulloblastoma, *miR-584-5p* inhibited the growth as well as potentiated the VCR response in U2OS osteosarcoma cells (Supplementary Fig. 1m). To test the specificity of anti-growth effect of *miR-584-5p*, we determined the effect of *miR-584-5p* on normal cells. *MiR-584-5p* did not affect the viability of neural stem cells as well as HEK-293T cells (Supplementary Fig. 1m–o).

### *MiR-584-5p* sensitizes VCR response in medulloblastoma

**Fig. 2:**
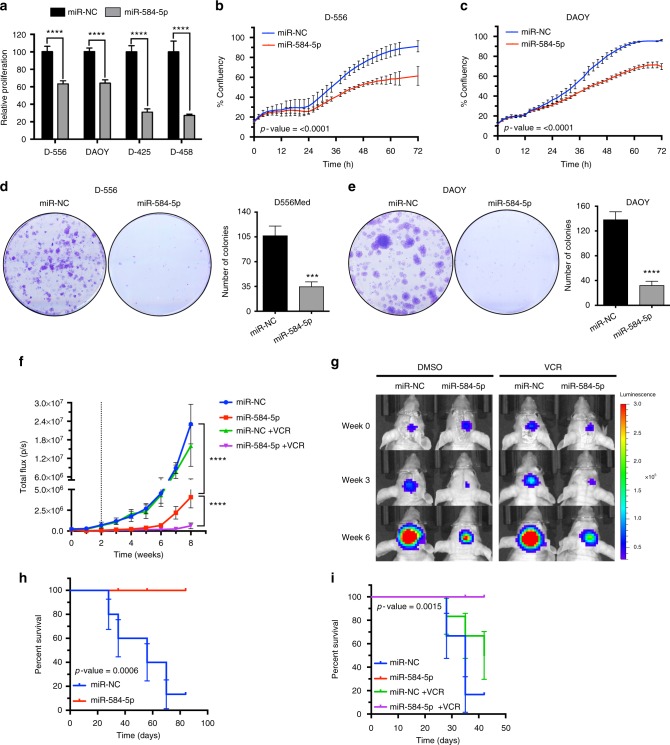
*MiR-584-5p* acts as a tumor suppressor and potentiates VCR response in vivo. **a** Cell proliferation in negative control miRNA (miR-NC) and *miR-584-5p* mimic-transfected MB cells. Cell proliferation was measured by CyQuant Cell Proliferation Assay. The *p*-values were determined using standard Student *t*-tests. Error bars represent mean ± SEM of three independent experiments (performed in sixtuplicate for each experiment). **b**, **c** Line graphs show cell proliferation of D556Med (**b**) and DAOY (**c**) cells transfected with miR-NC or *miR-584-5p* mimic. Cell proliferation was measured using an IncuCyte phase-only processing module. The *p*-values were determined by the sum-of-squares *F* test. Error bars represent mean ± SEM of three independent experiments (performed in triplicate for each experiment). **d**, **e** Clonogenic assay of D556Med (**d**) and DAOY (**e**) cells transfected with miR-NC or *miR-584-5p* mimic. Bar graph shows number of crystal violet-stained colonies. The *p*-value was calculated using a standard Student *t*-test. Error bars represent mean ± SEM of three independent experiments (performed in triplicate for each experiment). **f** Line graph shows tumor growth in athymic nude mice intracranially injected with DAOY–GFP–Luciferase cells transfected with miR-NC or *miR-584-5p* mimic and treated with vehicle control (dimethyl sulfoxide (DMSO); *n* *=* 12/group) or vincristine (VCR; *n* *=* 6/group). Dotted line represents the start of VCR injections. The *p*-values were determined by the sum-of-squares *F* test. Error bars represent mean ± SEM. **g** Live bioluminescence (BLI) images of mice intracranially injected with DAOY–GFP–Luciferase cells transfected with miR-NC or *miR-584-5p* mimic and treated with vehicle or VCR. **h**, **i** Kaplan–Meier survival curve of athymic nude mice intracranially injected with DAOY–GFP–Luciferase cells transfected with miR-NC or *miR-584-5p* mimic and treated with vehicle control (dimethyl sulfoxide (DMSO); *n* *=* 12/group) (**h**) or vincristine (VCR; *n* *=* 6/group) (**i**). The *p*-value was determined using the log-rank (Mantel–Cox) test. Error bars represent mean ± SEM ****, *p* < 0.0001; ***, *p* < 0.001

**Fig. 3:**
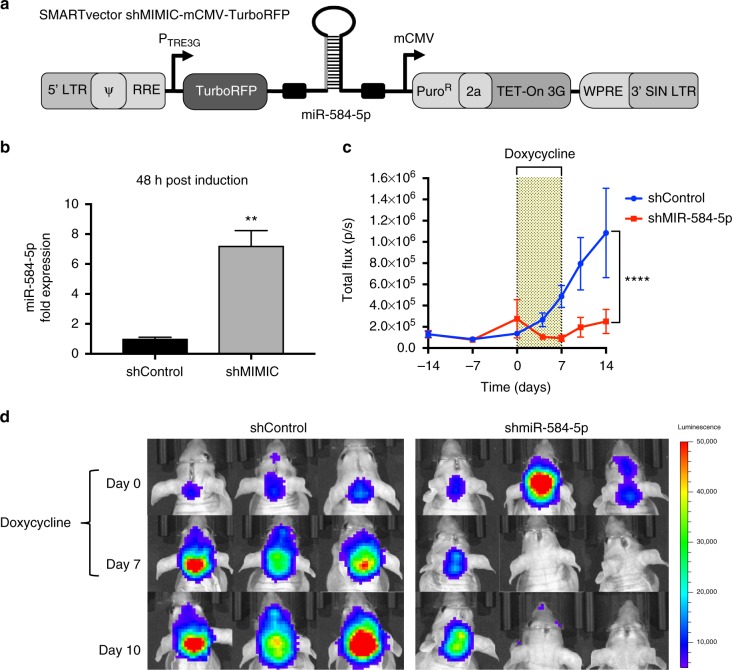
m*iR-584-5p* inhibits MB growth in vivo. **a** Schematic of inducible *miR-584-5p* lentiviral construct. **b** qPCR analysis of miR-584-5p levels in DAOY–GFP–Luciferase cells stably transfected with shcontrol mimic or *shmiR-584-5p* mimic-TurboRFP vector and treated with doxycycline using miR-584-5p-specific taqman probe. Error bars represent mean ± SEM of three independent experiments. The *p*-values were calculated using standard Student *t*-tests. **c** Line graphs showing tumor growth in athymic nude mice intracranially transplanted with DAOY–GFP–Luciferase cells stably transfected with inducible shcontrol mimic or sh*miR-584-5p* mimic-TurboRFP vectors (*n* *=* 7/group). Dotted line represents duration of doxycycline treatment to induce scrambled and *miR-584-5p* expression. The *p*-values were determined by the sum-of-squares *F* test. Error bars represent mean ± SEM. **d** Live bioluminescence (BLI) images of mice intracranially transplanted with DAOY–GFP–Luciferase cells transfected with inducible shcontrol mimic or sh*miR-584-5p* mimic-TurboRFP vector and treated with doxycycline

Next, we validated our in vitro results by determining the function of *miR-584-5p* in MB and by using in vivo tumor xenograft models to test the efficacy of *miR-584-5p*–VCR combination therapy. *MiR-584-5p* reduced the short-term and long-term viability of MB cell lines and also primary MB cells (Fig. 2a–e and Supplementary Fig. 2a–d). In addition, *miR-584-5p* inhibited the migratory capabilities of MB cells (Supplementary Fig. 2e). For in vivo studies, we performed orthotopic intracranial transplantation of the negative control miRNA (miR-NC) and *miR-584-5p*-transfected DAOY cells stably expressing green fluorescent protein (GFP)–luciferase. *MiR-584-5p* transfectant tumors showed significantly reduced MB growth in comparison with miR-NC-transfectant tumor (Fig. 2f, g) suggesting that *miR-584-5p* acts as a potent tumor suppressor in MB. Furthermore, combination therapy of *miR-584-5p* and a sub-lethal dose of VCR significantly reduced tumor growth in comparison with either *miR-584-5p* or VCR alone (Fig. 2f, g). Consistent with that finding, mice treated with *miR-584-5p* and *miR-584-5p*–VCR lived significantly longer than the miR-NC-transfectant group (Fig. 2h, i). To further substantiate those results, we tested the potency of miR-584-5p in inhibiting medulloblastoma growth using inducible miR-584-5p expression system. DAOY–GFP–luciferase cells were stably transfected with tet-on shControl and shMiR-584-5p expression vectors and treated with doxycycline to ensure induced expression of *miR-584-5p* (Fig. 3a, b). The tet-on shControl and shMiR-584-5p stably expressing cells were then intracranially implanted and after tumor reached a measurable size *miR-584-5p* expression was turned on by adding doxycycline to the drinking water. Induction of *miR-584-5p* expression resulted in significantly reduced medulloblastoma growth when compared with shControl medulloblastoma cells (Fig. 3c, d).

### *MiR-584-5p* target genes

**Fig. 4:**
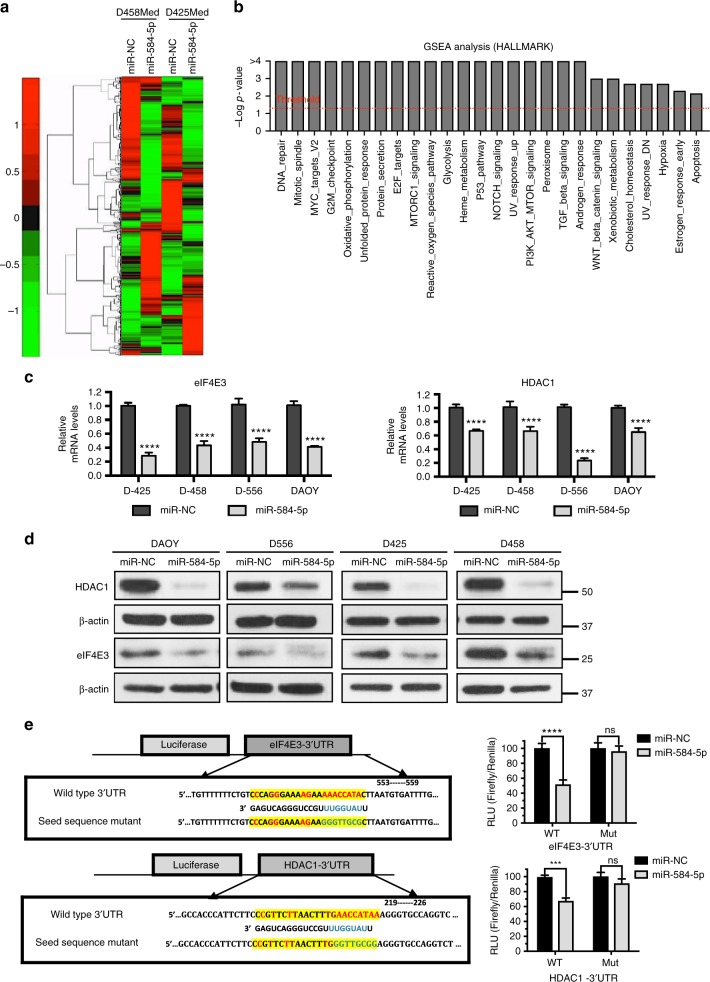
*MiR-584-5p* targets cancer, microtubule dynamics, and translation-associated genes. **a** Hierarchical clustering of gene expression changes in D425Med and D458Med cells treated with miR-NC or *miR-584-5p* mimic for 48 h. Data are *z*-transformed. The cutoff criteria for each gene are fold change > 2, sample intensity > 10, and *p*-value < 0.05. **b** Gene set enrichment analysis (GSEA) showing enriched biological processes in *miR-584-5p* mimic-treated MB cells. **c** qPCR analysis of eIF4E3 and HDAC1 expression in MB cells (D556Med, D425Med, D458Med, and DAOY) transfected with miR-NC or *miR-584-5p* mimic. The *p*-values were calculated using standard Student *t*-tests. Error bars represent mean ± SEM of three independent experiments (performed in triplicate for each experiment). **d** Western blot analysis of target genes in miR-NC or *miR-584-5p* mimic-transfected D556Med, D425Med, D458Med, and DAOY cells. Gel photographs are representative of at least three independent experiments. **e** Left, luciferase-eIF4E3/HDAC1 3′-UTR constructs. Inset boxes show *miR-584-5p*-binding sites in 3’-UTRs of eIF4E3 and HDAC1 (wild type). Also shown is *miR-584-5p* seed sequence mutated sequences. Right, relative luciferase activity in HEK293 cells transfected with miR-NC, *miR-584-5p*, or *miR-584-5p*-binding site mutant (mut). Values were normalized to firefly luciferase, which served as an internal control. The *p*-values were calculated using standard Student *t*-tests. Error bars represent mean ± SEM of three independent experiments. ****p < 0.0001; ***p < 0.001

To investigate the mechanism by which *miR-584-5p* inhibits MB growth and enhances VCR response, we performed gene expression analysis on D458Med and D425Med MB cells overexpressing *miR-584-5p* mimic. Gene set enrichment (GSEA) and Ingenuity pathway analyses revealed some highly altered downstream biological processes in *miR-584-5p*-expressing MB cells: DNA repair, mitotic spindle, cell cycle, G2-M checkpoint, apoptosis, nervous system development and function, cancer and translation initiation (Fig. 4a, b and Supplementary Figs. 3a, b). Examples of genes associated with those biological pathways included HDAC1 and eIF4E3, genes that were among the highly downregulated genes in our gene expression analysis (Supplementary data file 1 and Supplementary Fig. 3c). We decided to focus on HDAC1 and eIF4E3 as multiple target prediction algorithms, including Target Scan and Diana micro-T-CDS, predicted that *miR-584-5p* would target HDAC1 and eIF4E3. Importantly, both these genes consistently showed significantly reduced expression at the transcript and protein levels in all medulloblastoma cell lines treated with *miR-584-5p* (Fig. 4c, d and Supplementary Fig. 3d). Furthermore, HDAC inhibitor is reported to inhibit the growth of c-myc amplified medulloblastoma and is considered to be a good candidate for treating medulloblastoma patients^18^. Importantly, we confirmed eIF4E3 and HDAC1 to be direct targets of *miR-584-5p* as the luciferase activity of a construct containing luciferase gene fused with the 3′-UTR of eIF4E3 or HDAC1 was significantly reduced in MB cells expressing *miR-584-5p* mimic in comparison with scrambled, while mutation of *miR-584-5p* seed sequences in 3′-UTR of HDAC1/eIF4E3 resulted in no change in luciferase activity in the presence of *miR-584-5p* mimic in comparison with miR-NC (Fig. 4e). To further support that finding, we determined the levels of HDAC1/eIF4E3 in *miR-584-5p*-overexpressing primary MB cells. *MiR-584-5p* overexpression resulted in significantly reduced levels of HDAC1/eIF4E3 in primary MB cells (Supplementary Fig. 3e). Next, to test the *miR-584-5p* specificity in regulating the expression of its target genes, we determined the effect of miR-584-3p. Unlike *miR-584-5p*, overexpression of *miR-584-3p* did not alter the expression of eIF4E3 and HDAC1 in MB cells (Supplementary Fig. 3f). To further confirm the specificity of *miR-584-5p*–eIF4E3 signaling axis in MB, we tested the expression of eIF4E1, which acts as an oncogene in several cancers^19^, in *miR-584-5p* treated cells. Our results showed that eIF4E1 levels did not show any change in the MB cells overexpressing *miR-584-5p* (Supplementary Fig. 3g). Furthermore, depletion or overexpression of eIF4E3 did not alter eIF4E1 levels suggesting that loss of eIF4E3 was not compensated by eIF4E1 in *miR-584-5p*-overexpressing MB cells (Supplementary Fig. 3f). In addition to eIF4E3, silencing of HDAC1 did not result in altered expression of eIF4E1 (Supplementary Fig. 3g).

**Fig. 5:**
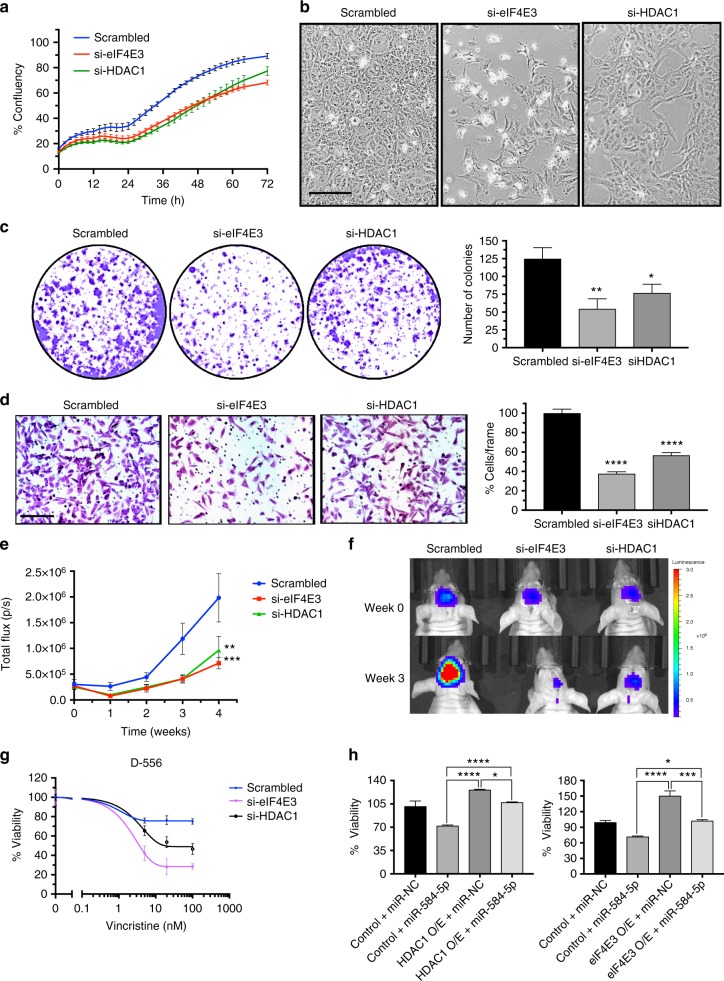
eIF4E3 and HDAC1 promote MB growth and inhibit VCR sensitivity of MB cells. **a** Proliferation of D556Med cell transfected with scrambled- or eIF4E3/HDAC1-siRNA. Cell proliferation was measured using an IncuCyte phase-only processing module. The *p*-values were determined by the sum-of-squares *F* test. Error bars represent mean ± SEM of three independent experiments. **b** Difference in morphology between D556Med cells transfected with scrambled-siRNA, eIF4E3-siRNA, or HDAC1-siRNA. **c** Colony-forming assay of D556Med cells transfected with scrambled-siRNA, eIF4E3-siRNA, or HDAC1-siRNA. Bar graph shows crystal violet-stained colonies counted microscopically. The *p*-value was calculated using a standard Student *t*-test. Error bars represent mean ± SEM of three independent experiments (performed in triplicate/experiment). **d** Photomicrograph showing migrated D556Med cells transfected with scrambled-siRNA, eIF4E3-siRNA, or HDAC1-siRNA. Bar graph shows number of migrated cells microscopically counted in ten different fields. The *p*-value was calculated using a standard Student *t*-test. Error bars represent mean ± SEM of three independent experiments. **e** Line graphs show mean tumor volume in nude mice intracranially injected with DAOY–GFP–luciferase cells transfected with scrambled-siRNA or eIF4E3/HDAC1-siRNA (*n* *=* 6 mice/group). The *p*-values were determined by the sum-of-squares *F* test. Error bars represent mean ± SEM. **f** Live bioluminescence (BLI) images of mice injected with DAOY–GFP–luciferase cells transfected with scrambled-siRNA or eIF4E3/HDAC1-siRNA (*n* *=* 6 mice/group). **g** VCR dose–response curve of D556Med cells transfected with scrambled-siRNA, eIF4E3-siRNA or HDAC1-siRNA and treated with VCR or vehicle. Cell viability was assessed using alamarBlue cell viability assay. The *p-*values were determined by the sum-of-squares *F* test. Error bars represent mean ± SEM of three independent experiments (performed in sixtuplicate/experiment). **h** *miR-584-5p* rescues cancer growth-promoting effects of eIF4E3 and HDAC1. Bar graphs showing viability of D556Med cells transfected with control plasmid or eIF4E3/HDAC1 expression vector or cotransfected with eIF4E3/HDAC1 expression vector and *miR-584-5p* mimic. The *p*-value was calculated using one-way analysis of variance (ANOVA) followed by Sidak’s multiple-comparisons test. Error bars represent mean ± SEM of three independent experiments (performed in sixtuplicate/experiment). ****p < 0.0001; ***p < 0.001; **p < 0.01. Scale bars: 50 μM (**b**, **d**)

To determine whether *miR-584-5p* imparts its tumor-suppressing and VCR-sensitizing effect by downregulating HDAC1/eIF4E3 levels in MB, we first investigated the function of eIF4E3 and HDAC1 in MB. Silencing HDAC1 or eIF4E3 led to significantly reduced short and long-term viability, proliferation, and migration of MB cells (Fig. 5a–d, Supplementary Figs. 4a, b). Furthermore, depletion of HDAC1 or eIF4E3 resulted in significantly reduced MB growth in orthotopic intracranial xenograft models (Fig. 5e, f). Moreover, depletion of HDAC1 or eIF4E3 significantly enhanced the VCR sensitivity in MB cell lines as well as in primary MB cells (Fig. 5g and Supplementary Fig. 4c). Notably, transfection of HDAC1/eIF4E3 expression construct resulting in increased expression of HDAC1 and eIF4E3 rescued the inhibition of viability and migration of MB cells elicited by *miR-584-5p* (Fig. 5h and Supplementary Figs. 5a, b). In addition, HDAC1/eIF4E3 overexpression blocked the VCR-sensitization effects of miR-584-5p in MB cells (Supplementary Fig. 5c). Taken together, our results suggested that *miR-584-5p* inhibits MB growth and progression by inhibiting the tumor-promoting function of HDAC1 or eIF4E3.

### *MiR-584-5p*–HDAC1/eIF4E3 regulates cell cycle progression

**Fig. 6:**
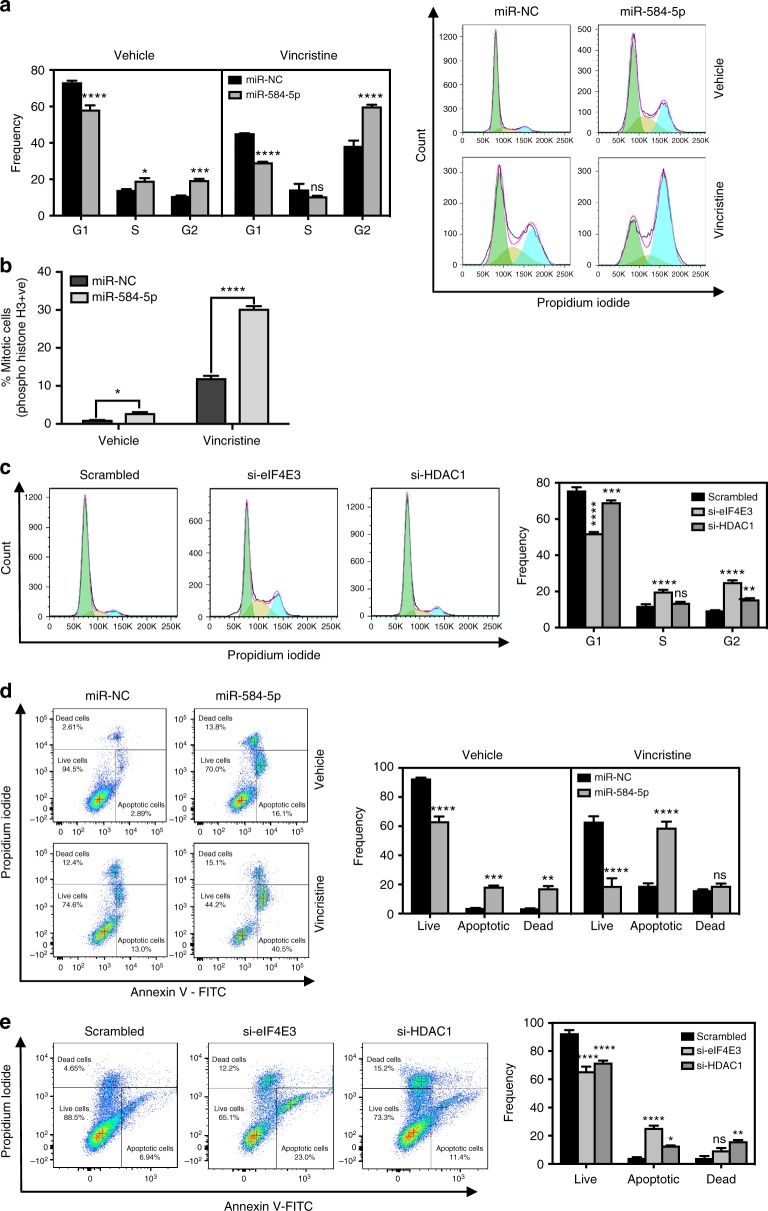
*MiR-584-5p*–HDAC1/eIF4E3 signaling regulates cell cycle progression of MB cells. **a** Bar graphs show quantification of DNA histograms (using FlowJo software) reflecting cell cycle progression in D556Med cells transfected with miR-NC or *miR-584-5p* and treated with vehicle or vincristine (VCR). DNA histograms were obtained from FACS analysis of cells treated with miR-NC or *miR-584-5p* and treated with vehicle or vincristine (VCR). Error bars represent mean ± SEM of three independent experiments. **b** Bar graphs show FACS analysis of Histone H3 pSer10-positive D-556 cells transfected with miR-NC or *miR-584-5p* and treated with vehicle or VCR. The *p*-values were calculated using standard Student *t*-tests. Error bars represent mean ± SEM of three independent experiments. **c** FACS analysis of cell cycle progression in D556Med cells transfected with scrambled-siRNA or eIF4E3-siRNA or HDAC1-siRNA. Bar graphs reflect DNA histograms quantified using the FlowJo software. Error bars represent mean ± SEM of three independent experiments. **d** FACS analysis of Annexin V–FITC-positive cells in D556Med cells transfected with miR-NC or *miR-584-5p* and treated with vehicle or VCR. Bar graphs show dot plots quantified using the FlowJo software. Error bars represent mean ± SEM of three independent experiments. **e** FACS analysis of Annexin V–FITC-positive cells in D556Med cells transfected with scrambled-siRNA or eIF4E3-siRNA or HDAC1-siRNA. Bar graphs show dot plots quantified using the FlowJo software. Error bars represent mean ± SEM of three independent experiments. The *p*-values for **d**–**e** were determined by two-way ANOVA followed by Tukey’s multiple-comparisons test. ****p < 0.0001; ***p < 0.001; **p < 0.01; *p < 0.05

To gain more insight into the MB growth and VCR-sensitizing activities of *miR-584-5p*–eIF4E3/HDAC1 signaling, we first determined how *miR-584-5p* mimic affected cell cycle progression of MB cells. MB cells treated with *miR-584-5p* mimic predominantly showed G_2_/M cell cycle arrest (Fig. 6a and Supplementary Fig. 6). Next, we performed a more in-depth analysis to identify whether *miR-584-5p* induces cell cycle arrest specifically at G2 or M or at both stages. Cell cycle analysis on MB cells using antibody against phospho-serine-histone H3, which is a mitosis marker, revealed that number of cells at M stage was significantly increased following *miR-584-5p* treatment compared to scrambled control in both D-556 and DAOY cells (Fig. 6b and Supplementary Fig. 7a). In addition, *miR-584-5p*–VCR combination treatment further increased the percentage of cells at G2/M-phase in comparison with either VCR or *miR-584-5p* alone (Fig. 6a, b and Supplementary Fig 7a). Next, we determined whether *miR-584-5p*’s effect on cell cycle progression may be mediated via its target genes eIF4E3/HDAC1. Indeed, silencing either eIF4E3 or HDAC1 resulted in increased number of mitotic cells and G_2_/M phase arrest (Fig. 6c and Supplementary Fig. 7b). The % of cells in M-phase was lower than the % of cells in G2/M in *miR-584-5p* and eIF4E3/HDAC1-siRNA treated MB cells suggesting that *miR-584-5p* and its targets eIF4E3/HDAC1 affect both the G2 and M-phases of cell cycle in MB cells (Fig. 6 and Supplementary Fig. 7b). Those findings may partly explain the sensitizing effect of the *miR-584-5p*–VCR combination because VCR induces G_2_/M phase arrest^20^. Next, we tested whether *miR-584-5p* mimic or eIF4E3/HDAC1 silencing-induced cell cycle arrest led to apoptosis of MB cells. Annexin V staining followed by fluorescence-activated cell sorter (FACS) analysis revealed that *miR-584-5p* mimic or eIF4E3/HDAC1 silencing resulted in increased apoptosis of primary MB cells as well as MB cell lines (Fig. 6d, e and Supplementary Fig. 7c).

### *MiR-584-5p*–eIF4E3/HDAC1 regulates microtubule dynamics

**Fig. 7:**
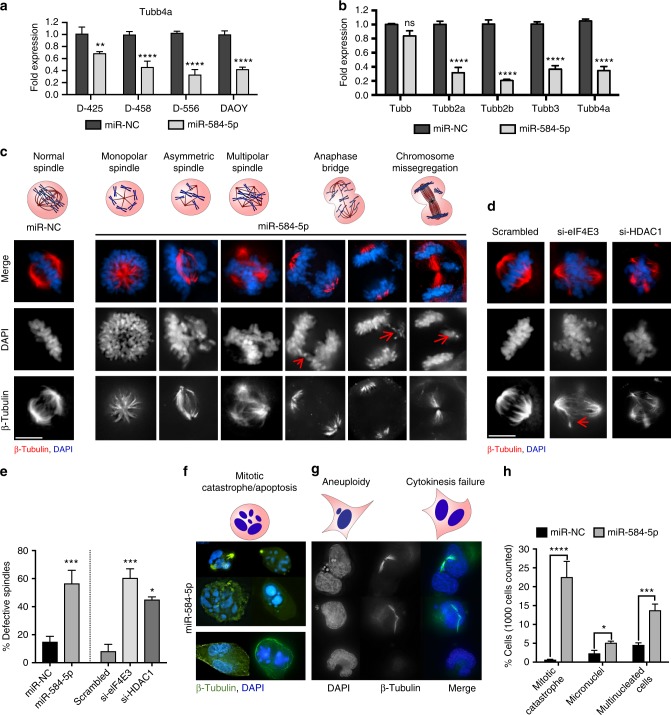
*MiR-584-5p*–eIF4E3/HDAC1 signaling regulates microtubule dynamics in MB cells. **a** qPCR analysis of TUBB4a in D556Med, D425Med, D458Med, and DAOY cells transfected with miR-NC or *miR-584-5p* mimic. The *p*-values were calculated using standard Student *t*-tests. Error bars represent mean ± SEM. **b** qPCR analysis of β-tubulin isotypes in D556Med cells transfected with miR-NC or *miR-584-5p* mimic. The *p*-values were calculated using standard Student *t*-tests. Error bars represent mean ± SEM of three independent experiments (performed in sixtuplicate). **c** Above, types of spindle defects. Below, immunofluorescence images of defective mitotic spindles in D556Med cells transfected with miR-NC or *miR-584-5p* mimic. DNA is stained with DAPI (blue), whereas spindle is stained with β-tubulin (red). Black and white images in the middle and lower panels are same as images shown in the top panel. Black and white images are included to clearly show *miR-584-5p* effects on chromatid organization (middle) and spindle defects (lower). **d** Immunofluorescence images of defective spindles in D556Med cells transfected with scrambled-siRNA or eIF4E3-siRNA or HDAC1-siRNA. Black and white images in the middle and lower panels are same as images shown in the top panel. Black and white images are included to clearly show the effects of eIF4E3 or HDAC1 silencing on chromatid organization (middle) and defects in spindles (lower). **e** Bar graphs show percentage of defective spindles in scrambled-siRNA, *miR-584-5p* mimic, eIF4E3-siRNA, or HDAC1-siRNA-transfected D556Med cells. The *p*-values were calculated using standard Student *t*-tests. Error bars represent mean ± SEM of three independent experiments. **f** Above, a cell undergoing mitotic catastrophe. Below, immunofluorescence images of *miR-584-5p-*transfected D556Med cells undergoing mitotic catastrophe. **g** Above, cells undergoing aneuploidy and cytokinesis failure. Below, immunofluorescence images of *miR-584-5p* mimic-transfected D556Med cells undergoing cytokinesis failure and exhibiting aneuploidy. **h** Bar graph shows number of D556Med cells transfected with miR-NC or *miR-584-5p* mimic undergoing mitotic catastrophe, aneuploidy, and cytokinesis failure. The *p*-values were calculated using standard Student *t*-tests. Error bars represent mean ± SEM of three independent experiments. ****p < 0.0001; ***p < 0.001; **p < 0.01; *p < 0.05. Scale bars: 10 μM (**c**, **d**, **f**, **g**)

VCR disrupts the integrity of the mitotic spindle and destabilizes microtubules^20^. Therefore, we wondered whether *miR-584-5p* and its target genes might affect microtubule dynamics. To investigate that, we first determined expression of tubulins because VCR resistance has been associated with altered expression of tubulin isotypes^21^. Our gene expression analysis revealed that TUBB4, a brain-specific β-tubulin, was among the highly downregulated genes, which real-time PCR analysis further confirmed (Fig. 7a). In addition to TUBB4, *miR-584-5p*-overexpressing cells consistently exhibited significantly reduced transcript levels of TUBB3, TUBB2a, and TUBB2b (Fig. 7b and Supplementary Fig. 8a). To further test how *miR-584-5p* and its target genes affect spindle integrity, we performed immunofluorescence analysis on *miR-584-5p*-overexpressing or eIF4E3/HDAC1-silenced MB cells. MB cells transfected with *miR-584-5p* mimic exhibited a variety of mitotic spindle defects, including monopolar, asymmetric, tripolar, and tetrapolar spindles as well as anaphase bridges (Fig. 7c, e and Supplementary Fig. 8b). In addition, *miR-584-5p* mimic treatment resulted in increased number of multinucleated cells, cells with mitotic catastrophe and micronuclei (Fig. 7f–h). Similarly, depleting eIF4E3 or HDAC1 resulted in spindle defects including multipolar as well as collapsed spindles in MB cells (Fig. 7d, e). Those findings suggested that *miR-584-5p* and its target genes eIF4E3 and HDAC1 are important regulators of microtubule dynamics.

### *MiR-584-5p* induces DNA damage and sensitizes IR response

**Fig. 8:**
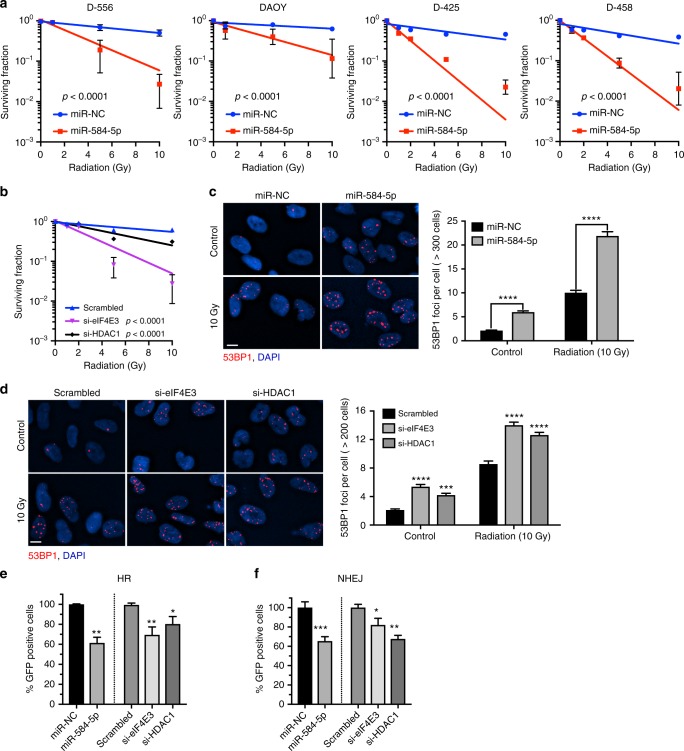
*MiR-584-5p* damages DNA and sensitizes radiation response in MB cells. **a** Ionizing radiation (IR) dose–response curves of D556Med, D458Med, D425Med, and DAOY cells transfected with miR-NC or *miR-584-5p* mimic and treated with increasing dose of IR. All values were normalized to 0 Gy. **b** IR dose–response curves of D556Med cells transfected with scrambled-siRNA or eIF4E3/HDAC1-siRNA. All values were normalized to 0 Gy. The *p-*values for **a** and **b** were determined by the sum-of-squares *F* test. Error bars in **a** and **b** represent mean ± SEM of three independent experiments. **c**, **d** Representative immunofluorescence images showing 53BP1 foci in D556Med cells transfected with miR-NC or *miR-584-5p* mimic (**c**) or scrambled-siRNA or eIF4E3/HDAC1-siRNAs (**d**) and treated with or without 10 Gy of IR. Bar graphs next to images show average number of 53BP1 foci/cell. The *p*-values were determined by two-way ANOVA followed by Sidak’s multiple-comparisons test. Error bars represent mean ± SEM of three independent experiments. **e** DR-GFP reporter assay. Flow cytometry analysis showing number of GFP-positive cells reflecting homologous recombination (HR) events. U2OS-DR-GFP cells were transfected with miR-NC or *miR-584-5p* or scrambled-siRNA or eIF4E3/HDAC1-siRNAs followed by transfection with pCAGGS vector with ISceI/GFP. I-Sce1 expression leads to double-strand breaks that HR repairs by using the wild-type GFP, resulting in GFP^+^ cells. The *p*-values were calculated using either Student *t*-tests (mimic) or one-way ANOVA followed by Dunnett’s multiple-comparisons test (siRNAs). Error bars represent mean ± SEM of three independent experiments. **f** EJ5-GFP reporter assay. Flow cytometry analysis showing number of GFP-positive cells reflecting non-homologous end joining (NHEJ) events. U2OS-EJ5-GFP cells were transfected with miR-NC or *miR-584-5p* or scrambled-siRNA or eIF4E3/HDAC1-siRNAs followed by transfection with pCAGGS vector with ISceI/GFP. I-Sce1 expression leads to double-strand breaks that NHEJ repairs, resulting in GFP^+^ cells. The *p*-values were calculated using either Student *t*-tests (mimic) or one-way ANOVA followed by Dunnett’s multiple-comparisons test (siRNAs). Error bars represent mean ± SEM of three independent experiments. ****p < 0.0001; ***p < 0.001; **p < 0.01; *p < 0.05. Scale bars: 10 μM (**c**, **d**)

VCR is routinely used during and after radiation therapy to treat MB patients^22^, mainly because microtubule-targeting agents such as VCR enhances the toxic effects of DNA-damaging agents, including radiation^23,24^. Moreover, cells in G_2_/M phase are the most sensitive to IR^25^. Our results showing *miR-584-5p* mimic causing mitotic defects suggest that *miR-584-5p* may also regulate radiosensitivity of MB cells. Indeed, *miR-584-5p* mimic-transfected MB cells were significantly more sensitive to IR than miR-NC-transfected cells (Fig. 8a). Next, we addressed whether eIF4E3 or HDAC1 may mediate *miR-584-5p*’s IR response. Indeed, silencing eIF4E3 or HDAC1 resulted in significantly increased IR response in MB cells (Fig. 8b).

To understand how *miR-584-5p* and its target genes may potentiate IR response, we assessed whether *miR-584-5p* or silencing eIF4E3/HDAC1 affected DNA damage repair in MB cells as IR damages DNA. To explore that, we assessed the amount of DNA strand breaks in *miR-584-5p* mimic or eIF4E3/HDAC1-silenced MB cells by determining the levels of p53 binding protein (53BP1) nuclear foci^26^. Immunofluorescence analysis showed significantly more 53BP1 foci in MB cells transfected with *miR-584-5p* mimic or eIF4E3/HDAC1-siRNA than in scrambled-transfected MB cells (Fig. 8c, d). Because *miR-584-5p* induces apoptosis, we reasoned that DNA strand break induced by *miR-584-5p* and eIF4E3/HDAC1 silencing may not be properly repaired. To confirm those findings, we performed functional assays to monitor non-homologous end joining (NHEJ) and homologous recombination (HR). NHEJ plays a critical role in repairing IR-induced DNA breaks in G1, S and G2 phases^27,28^. Furthermore, HR is also known to repair IR-induced double-strand breaks in G_2_ phase^29^. We used the I*Sce*I-based GFP reporter assays, which measure the frequency of double-strand break repair by NHEJ or HR^30–32^. FACS analysis showed significantly fewer GFP-positive cells in cells transfected with *miR-584-5p* mimic suggesting that *miR-584-5p* inhibits both NHEJ and HR-mediated DNA repair events (Supplementary Fig. 8c, d and Fig. 8e, f). Next, we tested whether HDAC1 and eIF4E3 may mediate miR-584-5p effects on NHEJ and HR. Silencing of eIF4E3 or HDAC1 inhibited both HR-dependent repair as well as NHEJ-dependent repair, suggesting that *miR-584-5p* inhibits, whereas eIF4E3 and HDAC1 support MB cells’ ability to repair DNA (Fig. 8e, f).

### *MiR-584-5p*–eIF4E3/HDAC1 regulates checkpoint functions

**Fig. 9:**
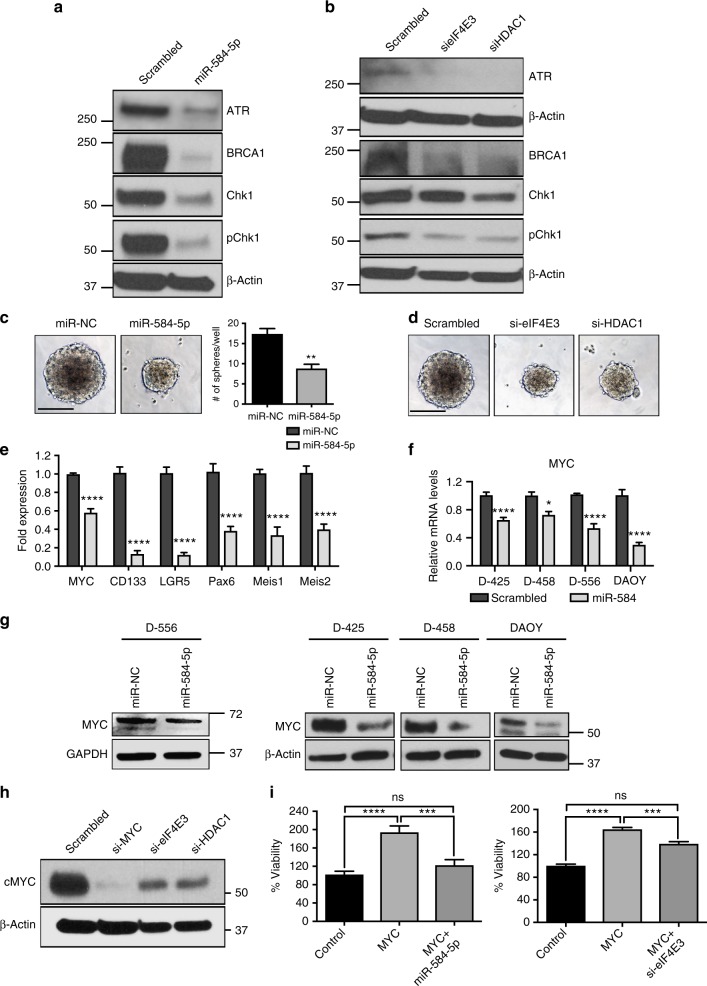
*MiR-584-5p*–eIF4E3/HDAC1 regulates c-MYC and MB stem cell proliferation. **a**, **b** Western blot analysis of DAOY cells treated with miR-NC or *miR-584-5p* mimic (**a**) or scrambled or eIF4E3/HDAC1-siRNA (**b**) using antibodies against indicated proteins. Gel pictures are representative of three independent experiments. **c** Medullospheres grown from D556Med cells transfected with miR-NC or *miR-584-5p* mimic. Bar graphs show number of medullospheres obtained from D556Med cells transfected with miR-NC or *miR-584-5p* mimic. The *p-*value was calculated using a standard Student *t*-test. Error bars represent mean ± SEM of three independent experiments (performed in triplicate for each experiment). **d** Medullospheres grown from D556Med cells transfected with scrambled-siRNA or eIF4E3/HDAC1-siRNAs. **e** qPCR analysis of stem cell markers in D556Med cells transfected with miR-NC or *miR-584-5p* mimic. The *p*-value was calculated using one-way ANOVA followed by Sidak’s multiple-comparisons test. Error bars represent mean ± SEM of three independent experiments (performed in triplicate for each experiment). **f** qPCR analysis of MYC expression in D556Med, D425Med, D458Med, and DAOY cell lines. The *p*-values were calculated using standard Student *t*-tests. Error bars represent mean ± SEM of three independent experiments (performed in triplicate for each experiment). **g** Western blot analysis of c-Myc in D556Med, DAOY, D425Med, and D458Med cells transfected with miR-NC or *miR-584-5p* mimic. **h** Western blot analysis of MYC in D556Med cells transfected with siRNA against scrambled, eIF4E3, HDAC1, or c-Myc. Gel pictures in **g** and **h** are representative of three independent experiments. **i** *miR-584-5p* and eIF4E3 rescue c-Myc-dependent MB cell growth. Bar graphs show percentage of cell viability in control vector, c-Myc expression vector, or *miR-584-5p* mimic plus c-Myc expression vector or eIF4E3-siRNA plus c-Myc expression vector*-*transfected D556Med cells. Cell viability was assessed using alamarBlue. The *p*-value was calculated using one-way ANOVA followed by Sidak’s multiple-comparisons test. Error bars represent mean ± SEM of three independent experiments (performed in sixtuplicate for each experiment). ****p < 0.0001; ***p < 0.001; **p < 0.01; *p < 0.05. Scale bars: 100 μM (**c**, **d**)

Having shown that *miR-584-5p* not only induces DNA damage but also induces cell cycle arrest and mitotic defects, we sought to understand the molecular mechanism by which *miR-584-5p* may mediate those events. To explore that, we examined the status of checkpoint proteins/mediators that regulate the cell cycle progression and mitotic entry to prevent the segregation of damaged chromosomes. Furthermore, both NHEJ and HR are regulated by ATR/ATM-dependent checkpoint control^33,34^. *MiR-584-5p* mimic-transfected MB cells showed significantly lower levels of activated CHK1 and ATR as well as BRCA1, which affects how long the G_2_/M checkpoint phase lasts (Fig. 9a and Supplementary Fig. 8e). Next, we determined whether *miR-584-5p* target genes eIF4E3 and HDAC1 may also regulate levels of checkpoint proteins. Depletion of eIF4E3 or HDAC1 resulted in reduced levels of CHK1, pCHK1, ATR, and BRCA1 (Fig. 9b and Supplementary Fig. 8f). These findings indicate that *MiR-584-5p* regulates checkpoint functions by targeting eIF4E3 and HDAC1.

### *MiR-584-5p* inhibits MB stem cell proliferation

Several research groups have posited the presence of cancer stem-like cells in MBs^35^. Moreover, the cancer stem-like cells in general have not responded to radiation and chemotherapy drugs^35^. Therefore, we wondered whether *miR-584-5p* may inhibit MB stem-like cell self-renewal/proliferation. Consistent with the characteristics of cancer stem-like cells, MB cells cultured in serum-free stem cell media formed tumor spheres that were serially passaged for many cycles (Fig. 9c and Supplementary Fig. 9a). *MiR-584-5p* mimic-transfected MB cells formed significantly smaller and fewer spheres (Fig. 9c and Supplementary Fig. 9a). Silencing *miR-584-5p* target genes eIF4E3 and HDAC1 also inhibited MB stem cell proliferation (Fig. 9d). Consistent with that finding, levels of several neural stem cell markers reportedly enriched in MB stem cells were significantly reduced in *miR-584-5p* mimic-transfected cells (Fig. 9e). Those findings are highly significant because, to our knowledge, this study is one of the first to propose a role for *miR-584-5p* and its target genes in regulating MB stem-like cell proliferation.

### *MiR-584-5p*–eIF4E3 signaling regulates c-Myc levels in MB

Our results showed that *miR-584-5p* overexpression or depletion of its target genes, eIF4E3/HDAC1, resulted in reduced growth/invasion and VCR/IR sensitization of c-Myc-amplified MB cells. Because c-Myc supports cancer stem cell growth, promotes radioresistance^36^, and has elevated levels in VCR-resistant cancer cells^37^, we asked whether *miR-584-5p* and its target genes may regulate c-Myc-amplified MB growth and therapy sensitization by targeting c-Myc levels/functions. Indeed, MB cells transfected with *miR-584-5p* mimic showed significantly reduced expression of c-Myc (Fig. 9f, g). Because no predicted *miR-584-5p*-binding site exists in the c-Myc 3′-UTR, *miR-584-5p* probably regulates c-Myc levels indirectly. To test that possibility, we asked whether *miR-584-5p* target genes may regulate c-Myc levels in MB. Moreover, eIF4E1 (not eIF4E3) induced c-Myc protein expression in diffuse B-cell lymphoma cells^38^. Surprisingly, our results revealed that depletion of eIF4E3 and HDAC1 significantly reduced c-Myc protein levels (Fig. 9h and Supplementary Fig. 9b). The pronounced effect of HDAC1 inhibition on c-Myc levels in D556Med than that of DAOY cells is likely because D556Med is c-Myc amplified, while c-Myc though expressed but is not amplified in DAOY cells. Next, we addressed whether *miR-584-5p* inhibits MB growth in part by targeting the eIF4E3–c-Myc signaling cascade. C-Myc overexpression rescued the effects of *miR-584-5p* on MB growth and progression (Fig. 9i and Supplementary Fig. 9c). Furthermore, MB cells transfected with c-Myc expression vector followed by eIF4E3-siRNA rescued the growth-promoting effect of c-Myc overexpression (Fig. 9i and Supplementary Fig. 9D). Those results suggest that eIF4E3 and c-Myc constitute a positive feedback loop to support MB growth and that *miR-584-5p* plays an important role in regulating the eIF4E3–c-Myc oncogenic axis in MB.

### *MiR-584-5p* expression is significantly lower in MB

**Fig. 10:**
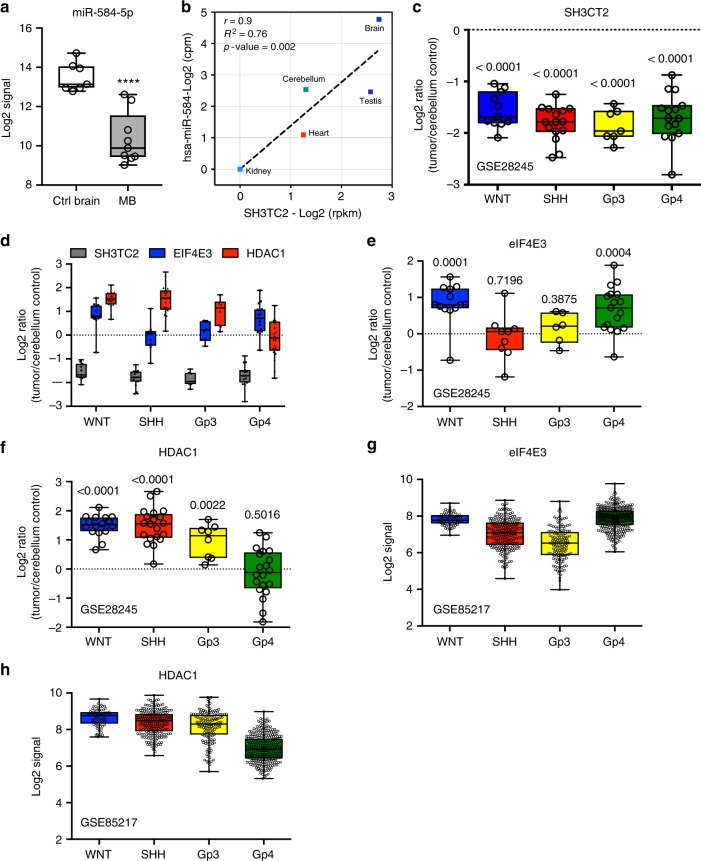
Lower *miR-584-5p* expression parallels with higher eIF4E3/HDAC1 levels in MB. **a** Meta-analysis of GSE42657 data set showing *miR-584-5p* expression levels in human MB samples and control cerebellum. Data represent Log2 ratio of tumor/cerebellum. **b** Meta-analysis of miRIAD: intragenic microRNA database showing correlation between *miR-584-5p* expression and the expression of its host gene SH3TC2. **c** Meta-analysis of GSE28245 data set showing SH3TC2 expression levels in different sub-types of MB samples (WNT: *n* = 16; SHH: *n* = 20; Gp3: *n* = 20; Gp4: *n* = 20) and control cerebellum. Data represent Log2 ratio of tumor/cerebellum control. *p*-values are calculated using the Wilcoxon-signed rank test (theoretical median = zero). **d** Meta-analysis of GSE28245 data set showing SH3TC2, eIF4E3, and HDAC1 expression levels in different sub-types of MB. Data represent Log2 ratio of tumor/cerebellum control. **e**, **f** Meta-analysis of GSE28245 data set showing eIF4E3 (**e**) and HDAC1 (**f**) expression levels in different sub-types of MB. Data represent Log2 ratio tumor/cerebellum control. *p*-values are calculated using the Wilcoxon-signed rank test (theoretical median = zero). **g**, **h** Meta-analysis of GSE85217 data set showing eIF4E3 (**g**) and HDAC1 (**h**) expression levels in different sub-types of medulloblastoma. Data represent Log2 ratio of tumor/cerebellum control (WNT: *n* = 763; SHH: *n* = 223; Gp3: *n* = 144; Gp4: *n* = 326. Whiskers in the box plots represent minimum to maximum value. The center line in the box represents the median value. Black open circles in the box represent individual MB patient tumor sample. ****p < 0.0001; ***p < 0.001; **p < 0.01

Next, we determined whether *miR-584-5p* and its target genes, eIF4E3 and HDAC1, show inverse expression pattern in MB. Meta-analysis of a data set from GSE42657^39^ showed that *miR-584-5p* expression is significantly lower in MB in comparison with normal control (Fig. 10a). Since there is no large publicly available data set for *miR-584-5p* expression, we determined the expression level of the host gene SH3CT2 that harbors *miR-584-5p*, in medulloblastoma samples. We used SH3TC2 gene as a proxy for *miR-584-5p* as expression of both SH3TC2 and *miR-584-5p* is driven by the same promoter and our analysis of the publicly available data on miRIAD database^40^ showed a strong correlation (*r* = 0.9, *R*^2^ = 0.76 and *p*-value = 0.02) between *miR-584-5p* and its host gene expression in different human tissues with highest expression and most robust correlation in the brain and cerebellum (Fig. 10b). Meta-analyses of Gene expression omnibus data sets GSE28245^41^ (*n* = 64) and GSE85217^42^ (*n* = 763) data sets revealed that SH3CT2 expression was markedly lower, while expression of eIF4E3 and HDAC1 was higher in all sub-types of medulloblastoma (Fig. 10c–h and Supplementary Fig. 10a). To further confirm those results, we examined *miR-584-5p* expression in MB patient-derived xenografts. The expression level of *miR-584-5p* was significantly lower in MB patient-derived xenografts than in induced pluripotent stem cell (iPSC)-derived neural progenitor cells and differentiated neurons (Supplementary Fig. 10b).

## Discussion

The existing therapeutic approach to MB includes high doses of craniospinal radiation and chemotherapy. Though that approach has improved survival, 20–30% of MB patients still succumb. Furthermore, patients with high-risk (such as c-Myc amplified) and metastatic tumors have an extremely poor survival rate. Moreover, the debilitating side effects associated with radiation and chemotherapy severely compromise survivors’ quality of life. Those facts underscore the urgency of developing new therapeutic regimens for treating MB. We used an unbiased genomic screen to identify *miR-584-5p* as a potent tumor suppressor miRNA that inhibits MB growth and progression of MB in general and c-Myc-amplified MB in particular. In addition, using sub-lethal dose of VCR/IR and in vitro and in vivo *models*, we have shown that miR-584-5p improves the efficacy of VCR/IR in medulloblastoma cells. *MiR-584-5p* potentiates response to IR and VCR by damaging DNA and inducing spindle defects in MB cells. *MiR-584-5p* imparts its tumor-suppressing and therapy-potentiating effects by targeting eIF4E3, HDAC1, and c-Myc in MB (Supplementary Fig. 11). Since adverse side effects of VCR and IR are also known to be proportional to dosing^11,43^, our results suggest that if used as a therapeutic, *miR-584-5p* not only can inhibit high-risk MB growth but also can lower the VCR and radiation dose (and hence lower the associated toxic effects) required to kill MB cells.

We have shown that *miR-584-5p* target gene eIF4E3 supports MB growth and progression. In contrast to our results, Osborne et al. showed that eIF4E3 inhibited anchorage-independent growth of NIH3T3 fibroblast and U2OS osteosarcoma cells^15^. However, Landon et al. later proposed that eIF4E3 can also support cell viability by inducing the expression of pro-proliferative genes^38^. Furthermore, eIF4E3-induced n-Myc, whereas eIF4E1-induced c-Myc expression in diffuse large B-cell lymphoma^38^. In this study, we find that eIF4E3 induces c-Myc protein expression. Translation initiation factors can regulate how any specific mRNA, including oncogenic mRNAs, can be translated. Therefore, eIF4E3 hyperactivation may result in increased abundance and activity of c-Myc, which in turn can play a causal role in cellular transformation of neural precursor cells and support MB growth by promoting cell proliferation, blocking apoptosis, or both. Supporting that notion, c-Myc transcriptionally activates Mcl-1^44^, an anti-apoptotic protein that is often upregulated in cancers and promotes cancer cell survival.

We have shown that the *miR-584-5p*–eIF4E3/HDAC1 signaling axis regulates VCR sensitivity by regulating microtubule dynamics in MB. eIF4E3 and HDAC1 may affect the dynamics of microtubules by directly interacting with them. Supporting that notion, HDAC1 forms a complex with microtubules in pathological conditions associated with axonal damage^45^. Though no direct evidence exists that eIF4E3 interacts with microtubules, translation initiation factors including eIF3, eIF4E, and eIG4G co-fractionate with microtubules^46^. In addition, *miR-584-5p* may affect microtubule organization/polymerization by inhibiting eIF4E3–c-Myc signaling because c-Myc interacts with α-tubulin and polymerized microtubules^47^. Furthermore, a transcriptionally inactive form of myc called myc-nick promotes α-tubulin acetylation and microtubule stabilization^48^.

We show here that *miR-584-5p* suppresses MB growth by inhibiting checkpoint function and HR/NHEJ-mediated DNA repair. These findings suggest that *miR-584-5p* mimic-treated cells with unrepaired DNA damage enter mitosis. There, they incur *miR-584-5p*–induced spindle defects, resulting in mitotic catastrophe, an event that precedes cell death (Fig. 7f–h). Moreover, mitotic entry with unrepaired DNA as well as impaired spindle formation results in mitotic catastrophe. Supporting that finding, our immunofluorescence analysis of *miR-584-5p-*overexpressing MB cells showed many cells with multipolar metaphase/anaphase, spindles with unorganized poles, micronuclei, and aneuploidy—well-established effects and outcomes of induced mitotic catastrophe (Fig. 7f–h). In addition to those defects, we observed *miR-584-5p* mimic-treated MB cells in which cytokinesis failed, resulting in giant cells with abnormal nuclei (Fig. 7e–h). That effect may be due to prolonged mitotic delay leading to mitotic slippage, in which cells exit mitosis and form multinucleated cells in G_1_ phase.

Our results reveal that silencing HDAC1 or eIF4E3 inhibits MB growth and sensitizes IR response by promoting DNA damage and NHEJ as well as HR-mediated DNA repair. HDAC inhibitors decrease the expression of proteins involved in repairing chemotherapy drug-induced DNA damage^49^. For example, MS-275, which inhibits class I HDACs, including HDAC1, decreases RAD51 expression and blocks HR in melanoma cells^50^. In addition, HDAC1 interacts with proliferating cell nuclear antigen, which is required for HR^51^. Importantly, HDAC inhibitors inhibit heat shock protein 90 (hsp90) chaperone function, which in turn results in proteosomal degradation of its client proteins including BRCA1, ATR, and CHK1 and consequently leading to impairment of the DDR and DNA repair^52–54^. Consistent with that, HSP90 inhibitor is known to sensitize cancer cells to DNA damage. Those facts suggest that approaches aimed at inhibiting HDAC1 (such as *miR-584-5p*) may inhibit MB growth and progression as well as sensitize therapy response by affecting multiple aspects of DNA damage response in MB cells. In addition to HDAC1, *miR-584-5p* may affect DNA damage and repair in MB by targeting eIF4E3-dependent signaling. Increased expression of eIF4E3 in MB may increase translation of those mRNAs involved in cell survival and DNA damage response. For example, inhibiting eIF4G1 delays resolution of DNA double-strand breaks in breast cancer cells by reducing CHK1, CHK2, and BRCA1 levels^55^. eIF4E3 interacts with eIF4G1^56^. Furthermore, our results show that *miR-584-5p* inhibits CHK1 and ATR levels in MB cells.

We have shown that *miR-584-5p*–HDAC1/eIF4E3 is a new signaling axis that regulates growth, progression, and therapy response in MB (Supplementary Fig. 11). To our knowledge, ours is the first study that implicates *miR-584-5p*, eIF4E3, and HDAC1 in regulating microtubule dynamics and DNA damage response in MB. Furthermore, the VCR-potentiating and IR-potentiating effects of *miR-584-5p* mimic and eIF4E3/HDAC1 inhibition serve as a strong rationale for developing *miR-584-5p* mimic or inhibitors of eIF4E3/HDAC1 as therapeutic regimens for treating MB in general and c-Myc-amplified MB in particular. One of the challenges for treating MB using miRNA/siRNA therapeutics is inefficient delivery across the blood–brain barrier. There are several different delivery strategies that have been used and are currently being developed to deliver miRNAs across the BBB. One of the strategies successfully used to deliver miR-182 in glioblastoma model is spherical nucleic acids (SNA) consisting of gold nanoparticle. Intravenous injection of miR-182-SNAs was reported to cross blood–brain/blood–tumor barriers resulting in reduced tumor burden and increased survival of animals in orthotopic GBM xenograft model^57^. Thiol-cross-linked low molecular weight polyethylenimine (LMW PEI) conjugated with brain-targeting rabies virus glycoprotein is another approach that was used to deliver miR-124a with effective brain targeting^58^. In addition, physical/chemical disruption^59^, and convection-enhanced delivery approaches^60^ have been successfully tested to overcome the BBB. In one of the clinical trials, implantation of an ultrasound (US) transducer in the skull of GBM patients led to safe and reversible BBB opening and enabled the crossing contrast agent and carboplatin into the brain^61^. This study suggests that ultrasound-directed opening of BBB can also be used to deliver miRNA/siRNA across the BBB. Taken together, use of an optimized delivery vehicle will facilitate efficient delivery of *miR-584-5p* across the BBB and will facilitate its clinical development for treating MB.

## Methods

### Animals and intracranial xenografts

Housing and all experimental animal procedures were approved by the Institutional Animal Care and Research Advisory Committee of the University of Texas Health Science Center at San Antonio. Athymic nude mice (nu/nu) were obtained from Harlan, USA. Three- to 5-week-old athymic nude mice (nu/nu) were anesthetized using isoflurane by inhalation. A burr hole was drilled and using a Hamilton syringe, we then stereotactically implanted 1 million cells into the right corpus striatum at a depth of 3.5 mm at a point 2.5 mm lateral to the midline and 1.5 mm anterior to the bregma. After the surgery, the animal recovered on a heating pad in a normal mouse cage to maintain its body temperature at 37–37.2 °C. An ocular lubricating ointment (AKORN) was administered until spontaneous blinking resumed. Starting from week 2, control group animals received dimethyl sulfoxide, whereas VCR group animals received 0.5 mg of VCR (Sigma-Aldrich) per kg of body weight intraperitoneally once per week for 6 weeks. For tet-on inducible expression system, once tumor reached measurable size, miR-584-5p expression was induced by treating animals with 2 mg/ml of doxycycline in the drinking water starting week 2. The Xenogen Small-Animal Imaging System was used for subcellular imaging in live mice once per week. Animals were killed, and brains were fixed and analyzed.

### Cell lines and cell culture

D556Med, D425Med, and D485Med cell lines were a gift from Duke University. DAOY was obtained from the American Type Culture Collection (Manassas, VA, USA). Cells were cultured at 37 °C in a 5% CO_2_ humidified atmosphere in minimal essential medium plus 10% fetal calf serum, 1% nonessential amino acids, 1% l-glutamine, 100 IU of penicillin per ml, and 100 μg of streptomycin per ml. Human iPSC-derived neural stem cells and neurons were a gift from Dr. D. M. Lehman. DAOY–GFP–Luciferase cells were generated by transducing DAOY cells with lentivirus particles containing CMV-GFP-T2A-Luciferase Lentivector (A gift from Dr. Ratna Vadlamudi, UTHSCSA). A 99% pure population was sorted via Fluorescence-activated cell sorting of live cells using the Beckman Coulter MoFlo® Astrios™ cell sorter. For tet-on inducible system, DAOY–GFP–Luciferase-Tet-on-RFP-shMIMIC and shControl cells were generated by transducing DAOY–GFP-Luciferase cells with lentivirus particles containing shMIMIC Human Inducible microRNA hsa-miR-584-5p mCMV-TurboRFP or SMARTvector Inducible Non-targeting mCMV-TurboRFP lentivectors (Dharmacon™ # GSH11929-224648704 and VSC11499). To obtain a pure population, cells were subjected to puromycin selection (Gibco™, #A1113803). All cell lines were tested for mycoplasma. Primary MB cells (BT-28 and BT-50) were authenticated using STR analysis.

### MiRNA mimic library screen

The primary screen was conducted using MI00200 MISSION microRNA Mimic v.17, in which miRNA mimics were arrayed in a one-miRNA–one-well format in 96-well microtiter plates. Reverse transfection of 10^3^ D458Med cells was performed in triplicate. Forty-eight hours after transfection, cells were treated for 72 h with a 20% inhibitory concentration (2 nM) of VCR. Cell viability was assessed using CellTiter-Glo (Promega, Madison, WI, USA) according to the manufacturer’s protocol. *ath-miR-416* or *cel-miR-243* was added as a negative control, whereas siRNA against PLK-1 was used as a positive control in each plate. The effect of siPLK-1 and negative control miRNA controls was quantified using the Strictly Standardized Mean Difference (SSMD), which uses the mean and standard deviation of each control to statistically measure the dynamic range between positive to negative controls. An SSMD of ≥3 is considered to have very strong effect. Negative control microRNAs in VCR plates consistently scored 20% less viability than control plates. Raw signals from the entire screen were z-transformed to such that negative control microRNAs are around the 0. Candidate miRNAs were prioritized for validation by functional and interaction assays using *t*-tests (with pooled variance), false discovery rate (*Q* = 0.5%), and by using the magnitude of response (lowest 2.5 percentile of the distribution of the *μ*_VCR_/*μ*_Control_ ratios, i.e., more than 10% reduction in viability from miR-NC transfectant cells).

### MiRNA, siRNA, and plasmid transfection

*miR-584-5p* and miR-NC were purchased from Thermofisher; si-eIF4E3, si-HDAC1, si-MYC, and scrambled-siRNA were purchased from Sigma. MB cell lines were transfected using Lipofectamine RNAiMAX Reagent (Thermofisher) according to the manufacturer’s protocol. MiR-NC and *miR-584-5p* were used at 50 nM for all experiments unless otherwise indicated. eIF4E3 plasmid was purchased from Origene (#RC209694). HDAC1 (#13820) and c-Myc (#46970) plasmids were purchased from Addgene. MB cell lines were transfected using Lipofectamine 2000 (Thermofisher) according to the manufacturer’s protocol.

### Cell viability and proliferation assays

MB cells transfected with *miR-584-5p* mimic or target siRNAs were seeded in 96-well plates at a density of 5 × 10^3^ for D556Med and DAOY or 10 × 10^3^ for D425Med and D458Med per well for 72 h. Cell viability was assessed using CellTiter-Glo (Promega) or alamarBlue (Thermofisher), and proliferation was assessed using CyQuant Direct Cell Proliferation Assay (Thermofisher).

### Migration and colony formation assays

MB cells were transfected with *miR-584-5p*, miR-NC, scramble-siRNA, or target-siRNA (Sigma) for 24 h, harvested, and subjected to long-term clonogenic and migration assays^62^. For the long-term clonogenic assays, 1000 cells/well were reseeded in six-well plates for an additional 7–10 days until colonies were visible. Colonies were fixed with 4% paraformaldehyde and stained with 1% crystal violet. For the transwell migration assay, 100,000 cells were reseeded in Corning BioCoat Control Cell Culture Inserts with 8.0-µm PET Membrane in serum-free medium. Cells were allowed to migrate toward complete media for 24 h before fixation with 4% paraformaldehyde and staining with 1% crystal violet.

### Quantitative real-time PCR

Total RNA was extracted using miRNAeasy kit (Qiagen). Reverse transcription was done using either miScript II RT Kit (Qiagen) or *Taq*Man microRNA reverse transcription (Thermofisher). RT-qPCR was done using SYBR green (Qiagen) or *Taq*Man microRNA Control Assays according to manufacturer’s protocols. RT-qPCR primer sequences are listed in Table 1.

**Table 1:** List of primers

Name	Forward primer	Reverse primer
miR-584-5P	TTATGGTTTGCCTGGGACTGAG	miScript universal primer
eIF4E3	GCAAAGGGTGGCGTATGGAA	CCCGATGGTTGCTAACAGC
HDAC1	CTACTACGACGGGGATGTTGG	GAGTCATGCGGATTCGGTGAG
MYC	GGATTCCCGCCTCAGAATAAC	GTGGGTGTGGGTTGTTCAGG
TUBB^63^	CCCCATACATACCTTGAGGCGA	GCCAAAAGGACCTGAGCGAA
TUBB2A^63^	CTCAGATCAATCGTGCATCCTTAGTGAACTTCTGT	GTATAGATACCTTCACAGACAATACTGTAATTTTTAGACTTCCACA
TUBB2B^63^	ACGGGTTAGGGAAAGCGGA	TTCCGACACAAACGTTTATGTGA
TUBB3	GGCCAAGGGTCACTACACG	GCAGTCGCAGTTTTCACACTC
TUBB4	GGACAACTTCGTTTTCGGTCA	CCTTTCTCACAACATCCAGCAC

### Gene expression profiling

Total RNA was isolated from D425Med and D458Med cells after *miR-584-5p* transfection for 48 h. RNA samples were further processed at the UTHSCSA Genomics Core for gene expression profiling by using Illumina Human HT-12 v4 Expression BeadChip following the manufacturer’s standard protocol. Gene expression data were quantified and normalized (quantile normalization) using BeadStudio software (Illumina). A Union gene set of 1152 genes differentially expressed in both D425Med and D458Med cells treated with miR-NC or *miR-584-5p* mimic for 48 h was used. Data were *z*-transformed, and the cutoff criteria for each gene are a fold change > 2, sample intensity > 10, and *p*-value < 0.05.

### Flow cytometry analysis of cell cycle and apoptosis

MB cells were seeded in six-well plates and transfected with *miR-584-5p* mimic or target gene siRNAs and incubated for 48 h. Cells were then treated with VCR or vehicle for 72 h before being stained with propidium iodide and Annexin V–fluorescein isothiocyanate (FITC) to analyze apoptosis. For cell cycle analysis, treated cells were fixed using 70% ethanol for 24 h followed by propidium iodide staining. Cells were analyzed using either FACScantoII or LSRII cytometers, and data were quantified using FlowJo 10.2 software.

### Immunofluorescence and western blot analysis

We carried out immunofluorescence analyses on cells fixed in 4% paraformaldehyde as described previously^64^. We performed Western blot analysis on cell extracts from *miR-584-5p* mimic-transfected or target gene siRNA-transfected MB cell lines in accordance with previously reported studies^62,64^ (Supplementary Fig. 12). Antibodies against horseradish peroxidase-conjugated β-actin (1:50,000; #A5316), β-tubulin (#T8328), and glyceraldehyde-3-phosphate dehydrogenase (GAPDH) (1:25,000; #G9295) were purchased from Sigma-Aldrich. Antibody against eIF4E3 (1:1000; #17282-1-AP) was purchased from Proteintech. Antibodies against HDAC1 (1:1000; #5356), pCHK1 (1:1000; #2348), and CHK1 (1:1000; #2360) were purchased from Cell Signaling Technology (Danvers, MA, USA). Antibodies against MYC (1:1000; #Sc-40), BRCA1 (1:1000; #6954), and ATR (1:1000; #sc-515173) were purchased from Santa Cruz. Antibody against 53BP1 (1:300; #A300-272AT) was purchased from Bethyl Laboratories.

### Dual luciferase assay

Wild-type or mutant 3′-UTR segments of the eIF4E3 and HDAC1 gene were cloned downstream of the luciferase gene in pGL3-promoter vector at the SacI and SpeI restriction sites. HEK293 cells were cotransfected with *Renilla* luciferase vector (pRLnull 380) and firefly luciferase vector containing pGL3-wt–eIF4E3; pGL3-mut–eIF4E3; or pGL3-wt–HDAC1, pGL3-mut–HDAC1, or pGL3 overnight and incubated in fresh complete medium for an additional 48 h after transfection. Cells were then transfected with miRNA mimic (Invitrogen) for 24 h. Next, cells were harvested with 1× passive lysis buffer (Promega) and luciferase activity was read using GLOMAX 20/20 luminometer (Promega).

### Homologous recombination and non-homologous end joining assays

U2OS cells with stably integrated DR-GFP(HR) or EJ5-GFP constructs and the endonuclease ISceI expression vector were obtained from Dr. Maria Jasin and Dr. Jeremy Stark. cells were seeded in 12-well plate and transfected with microRNA, siRNAs or overexpression vectors or combinations. After 24 h, cells were transfected with ISceI expression vector. After 72 h, cells were harvested and GFP-positive cells were evaluated by flow cytometry on a BD FACS CANTO II flow cytometer. Data were analyzed using FlowJo 10.2 software.

### Statistical analysis

Statistical analysis was done using GraphPad Software, R, or Compusyn. Values and error bars in graphs represent the mean ± standard error of the mean (SEM). Respective n values are indicated in figure legends. *p*-values were determined by an appropriate statistical test like Student’s *t*-tests, analysis of variance (ANOVA) or sum-of squares tests. For studies involving mice, sample size is estimated by using *t*-test with mean difference between control and test groups. For example, for Fig. 2f, comparing control group of miR-584 alone vs test group miR-584 + VCR, assuming at the end of treatment, a log10-FLUX = 1 change with standard deviation about 0.5 (log10 unit), or effective size of 2, we estimated at least six mice are required to reach statistical power of 80% with significance at 0.05. We chose more mice for some experiments in order to observe differences in earlier time point during the treatment. Sample size calculations were performed using PASS 14 (NCSS, LLC, UTAH). For animal experiments, treatment groups were blinded and randomized.

## Electronic supplementary material

Supplementary Information

Description of Additional Supplementary Files

Supplementary Data 1

## Data Availability

The gene expression profiling data set for miR-584-5p overexpression in D425Med and D458Med cell lines is submitted to NCBI (GSE105059)^65^. The publically available data set miRIAD was used for showing correlation between miR-584-5p and SH3TC2; GSE28245, GSE42657 (and GSE85217 were used for analyzing SH3TC2, eIF4E3, and HDAC1 expression. The authors declare that all other relevant data supporting the findings of this study are available within this article, its supplementary information files, or upon request from the corresponding author.
