# Supplementary material for: MiR-584-5p potentiates vincristine and radiation response by inducing spindle defects and DNA damage in medulloblastoma
Source: Nat Commun. 2018 Oct 31;9:4541. doi: 10.1038/s41467-018-06808-8 (PMC6208371; doi:10.1038/s41467-018-06808-8)
Supplement: Supplementary file 1 — Supplementary Information [file 41467_2018_6808_MOESM1_ESM.pdf]

Supplementary Information

MiR-584-5p potentiates vincristine and radiation response by inducing spindle defects and DNA damage in medulloblastoma

Abdelfattah et. al.

Supplementary Figure 1

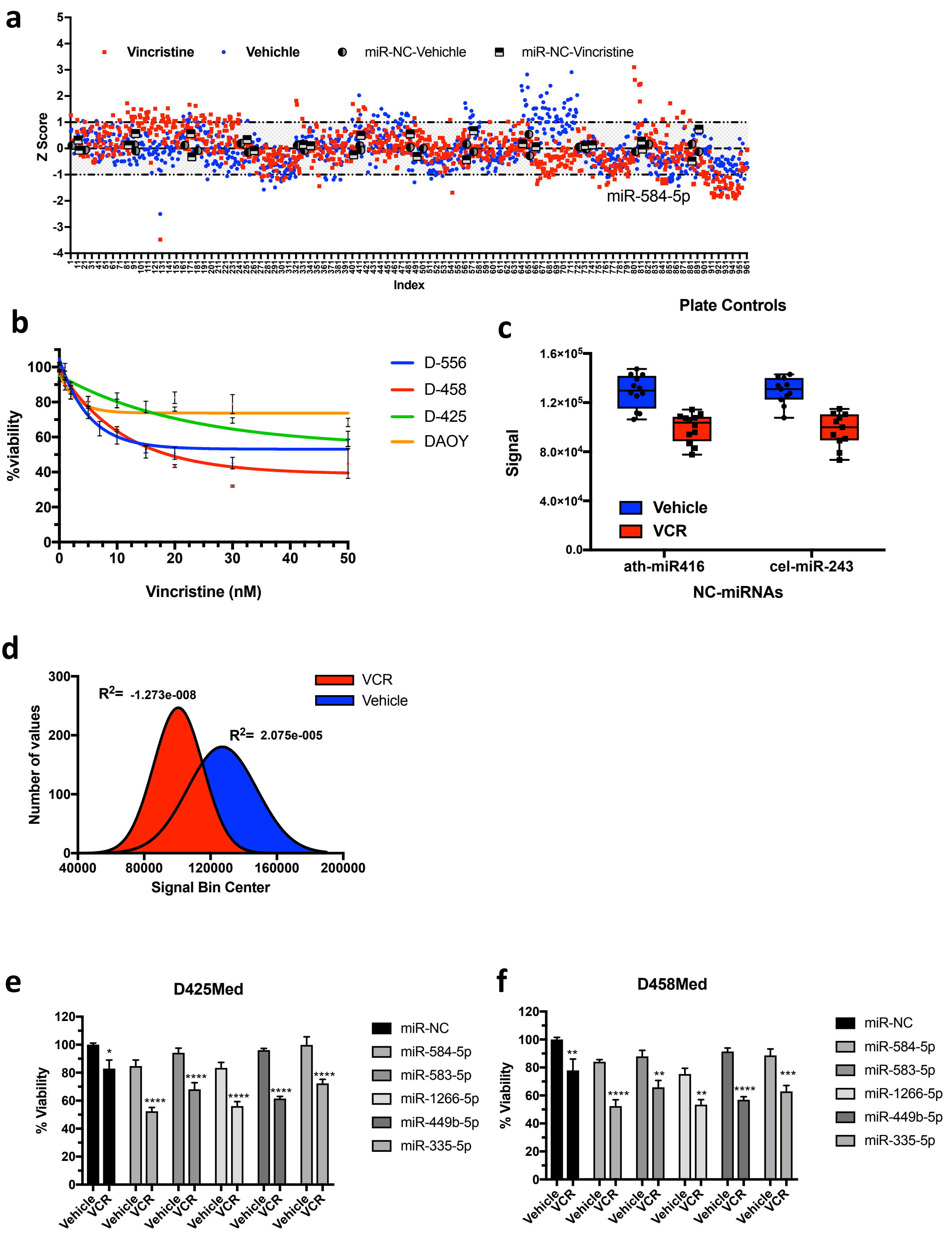

Supplementary Figure 1

g

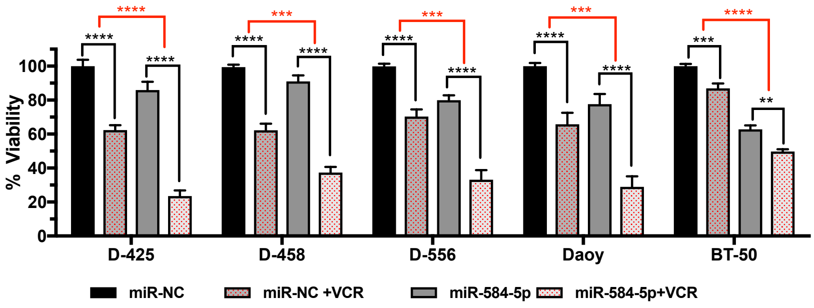

h

D-556		
VCR (nM)	miR-584-5p (nM)	Comb. Index
1.0	5.0	0.01542
2.0	10.0	0.03083
5.0	25.0	0.07708
10.0	50.0	0.15416
20.0	100.0	0.30831

i

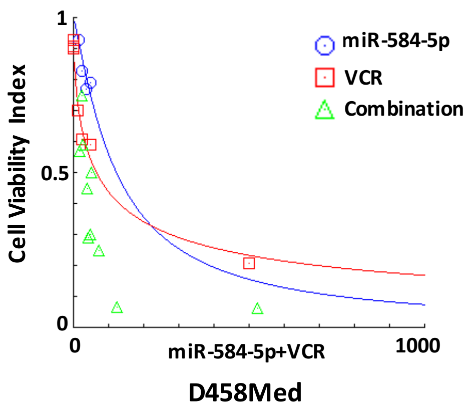

j

D458Med		
VCR (nM)	miR584 (nM)	CI
2.5	50.0	0.63834
5.0	50.0	0.3484
5.0	35.0	0.24304
5.0	75.0	0.28608
5.0	100.0	0.44671
40.0	50.0	0.16227
50.0	50.0	0.18974
100.0	50.0	0.19736
200.0	50.0	0.03914
1000.0	50.0	0.10047

k

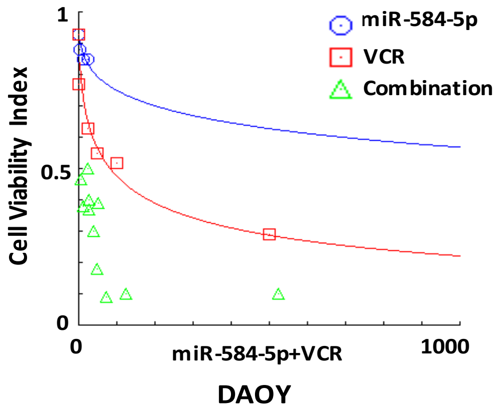

l

DAOY		
VCR (nM)	mir584 (nM)	CI
1.0	50.0	0.01747
5.0	50.0	0.01714
5.0	50.0	0.01304
5.0	10.0	0.02455
5.0	25.0	0.01284
5.0	100.0	0.01894
30.0	50.0	0.03459
50.0	50.0	0.01493
100.0	50.0	0.00606
200.0	50.0	0.01527
1000	50.0	0.07623

Supplementary Figure 1

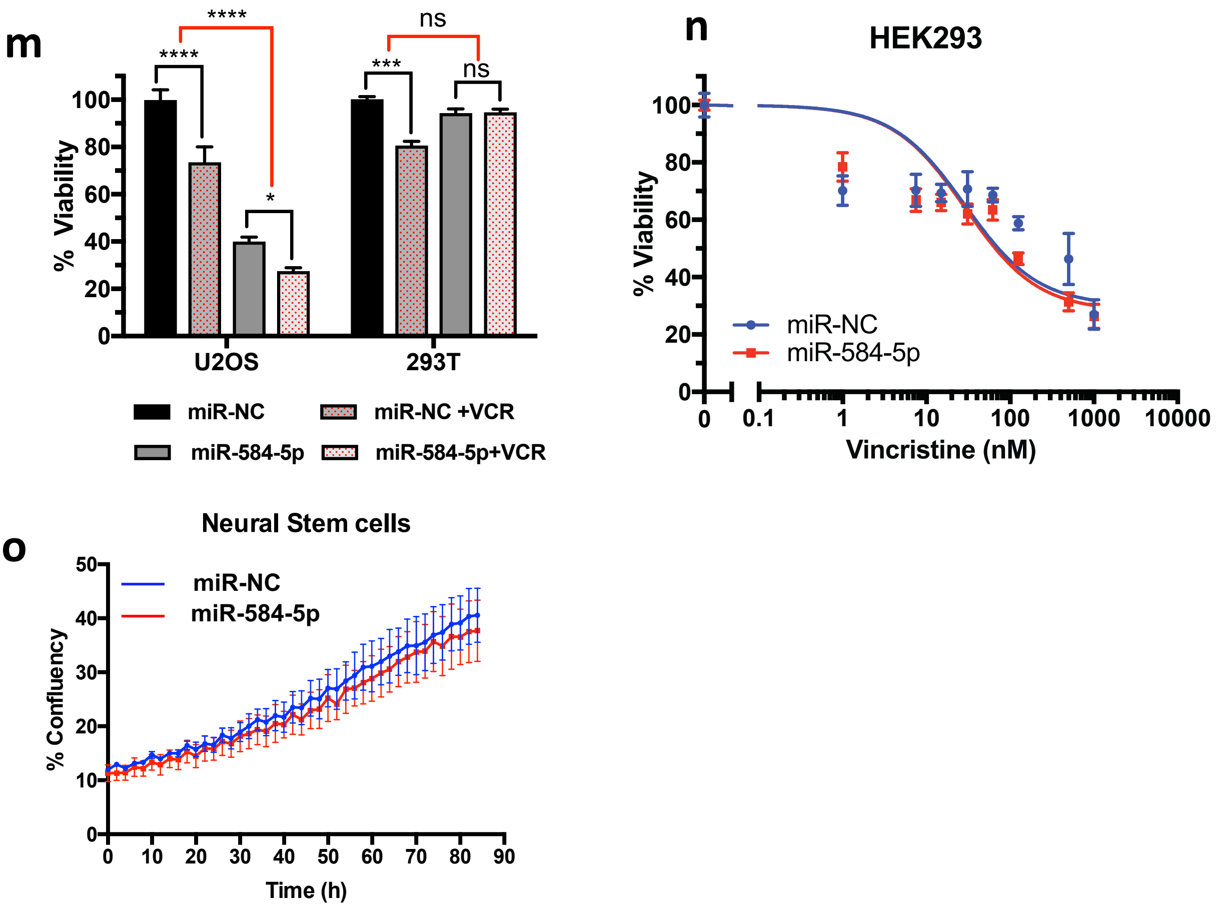

Supplementary Fig. 1. *MiR-584-5p* potentiates VCR response in MB without affecting normal cells. (a) Distribution of *z*-scores for D-458Med cells treated with vehicle (blue dots) or VCR (red dots). Cells transfected with negative control (NC) miRNAs (*ath-miR-416* or *cel-miR-243*) are represented by gray dots. (**b**) Line graphs show cell viability of D-556, D-425Med, D-458Med and DAOY cells treated with vehicle or increasing concentration of VCR. (**c**) Raw signal reflecting cell viability compared with NC miRNAs treated with vehicle or VCR cofirm 20% reduction in viability. (**d**) Analysis showing normal distribution of raw signal of primary screen plate controls in vehicle vs VCR plates. (**e, f**) Bar graphs show relative viability of D-425Med **(e)** and D-458Med **(f)** cells transfected with miR-NC or indicated miRNAs for 24 hours followed by treatment with vehicle or 2 nM of VCR for 72 hours. **(g)** Bar graphs show % viability of D-556Med, D-425Med, D-458Med, DAOY and BT-50 cells transfected with 50 nM of miR-NC or miR-584-5p mimic followed by 5 nM VCR treatment. **(h-l)** Synergistic effect of miR-584-5p with VCR. D556-Med (**h**), D458-Med (**i** and **j**) and DAOY (**k** and **l**) cells were treated with different concentrations of miR-584-5p and VCR before being subjected to cell viability assay. Compusyn software was used to calculate combination indices (CIs; **h**, **j** and **l**). **(m)** Bar graphs show % viability of U2OS and HEK293 cells transfected with miR-NC or miR-584-5p mimic treated with vehicle or 5 nM of VCR. p-Values for **e-m** were calculated using one-way ANOVA followed by Sidak's multiple comparisons test. **(n)** Dose–response curves showing effect of VCR in NC (blue line) and miR-584-5p mimic (red line)–transfected HEK293 cells. HEK293 cells were transfected with miR-NC or miR-584-5p mimic followed by treatment with VCR or vehicle for 72 hours. The p-value (p=0.08) was determined by the sum-of-squares F test. **(o)** Line graphs show proliferation measured using an IncuCyte of IPS-derived neural stem cells transfected with miR-NC or miR-584-5p. For **e-n**, error bars represent the mean ± SEM of atleast three independent experiments. ****P < 0.0001, *** P < 0.001; ** P<0.01; * P<0.05.

Supplementary Figure 2

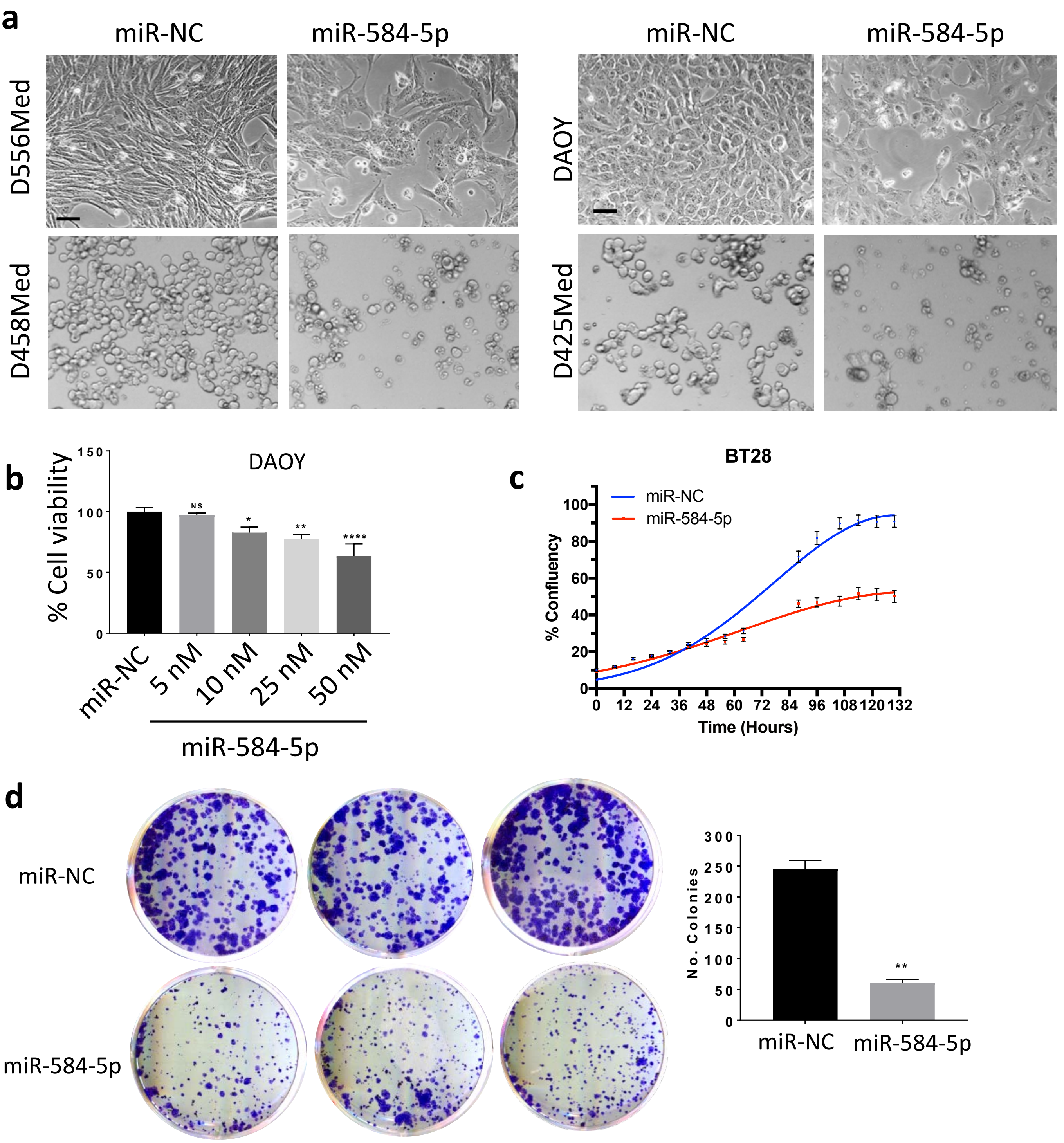

Supplementary Figure 2

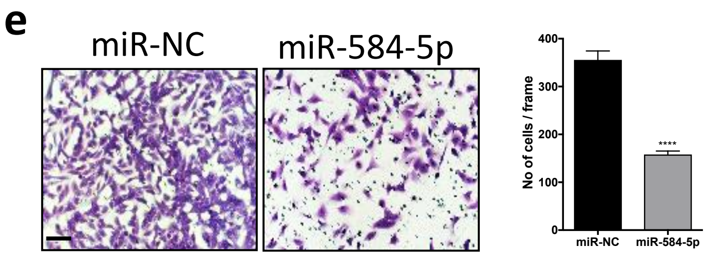

Supplementary Fig. 2. *MiR-584-5p* inhibits viability of MB cell lines and primary MB BT-28 cells. (a) Representative microscopic images showing the morphology and viability of D458Med, D425med, D556Med and DAOY cells transfected with miR-NC or *miR-584-5p*. Scale bar represents 50µM. **(b)** Bar graphs showing cell viability of DAOY cells transfected with miR-NC or increasing concentration of *miR-584-5p* mimic. Cell viability was assessed using alamarBlue® 48 hours after transfection. p-Value was calculated using one-way ANOVA followed by Sidak's multiple comparisons test. Error bars represent the mean of three independent experiments ± SEM**. (c)** Line graphs show proliferation measured using an IncuCyte phase-only processing module and reflected by % confluency of BT-28 cells transfected with miR-NC or *miR-584-5p* mimic. Error bars represent the mean ± SEM of three independent experiments. (**d**) Clonogenic assay of primary BT-28 cells transfected with miR-NC or *miR-584-5p* mimic. Bar graph shows number of crystal violet–stained colonies. The *p*-value was calculated using a standard Student *t*-test. Error bars represent mean ± SEM of three independent experiments (performed in triplicate for each experiment). **(e)** Photomicrograph shows number of migrated D556Med cells transfected with miR-NC or *miR-584-5p* mimic. Scale bar represents 50µM. Bar graph shows number of migrated cells counted microscopically in at least 10 fields. The *p*-value was calculated using a standard Student *t*-test. Error bars represent mean ± SEM of three independent experiments. ****P < 0.0001.

Supplementary Fig 3

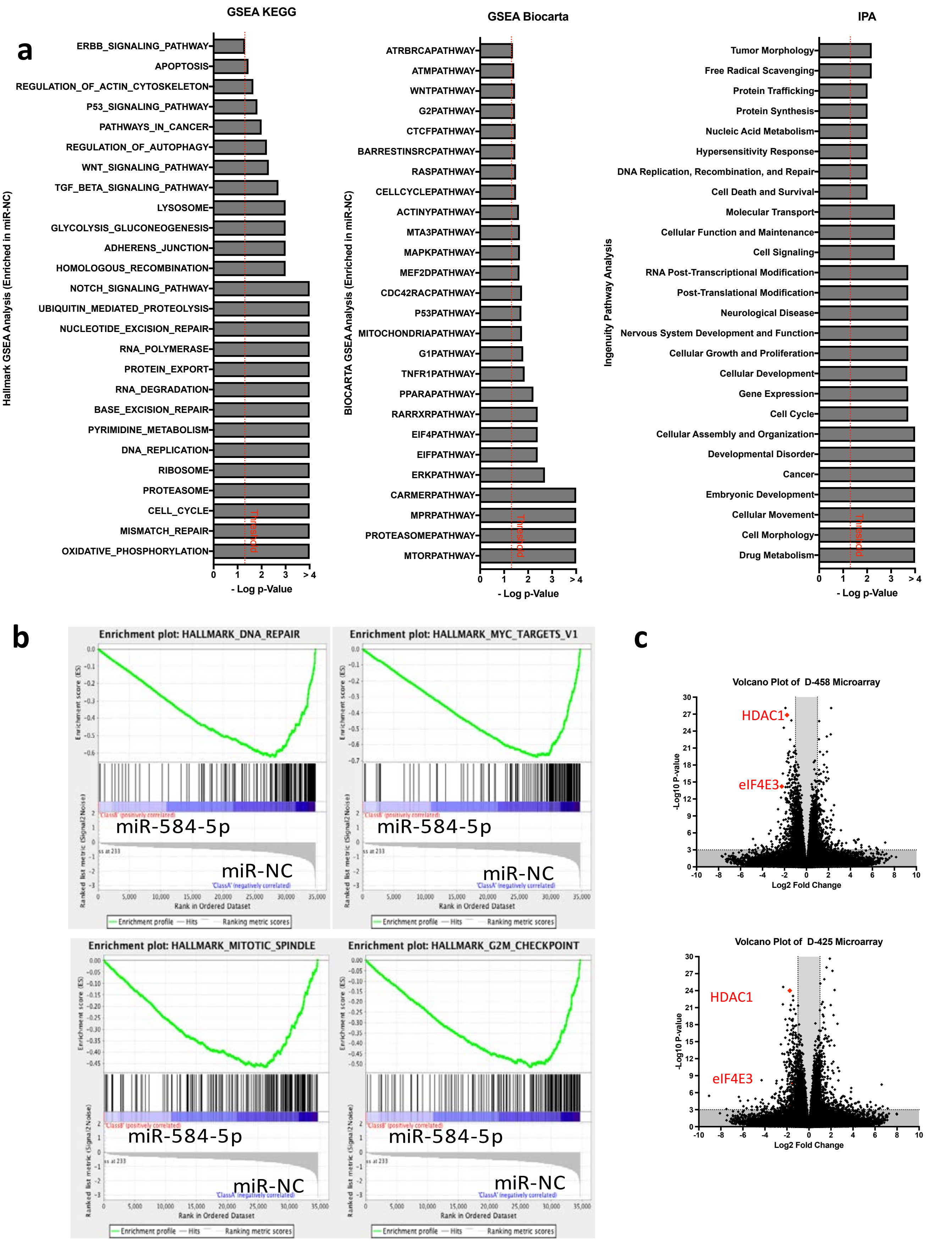

Supplementary Fig 3

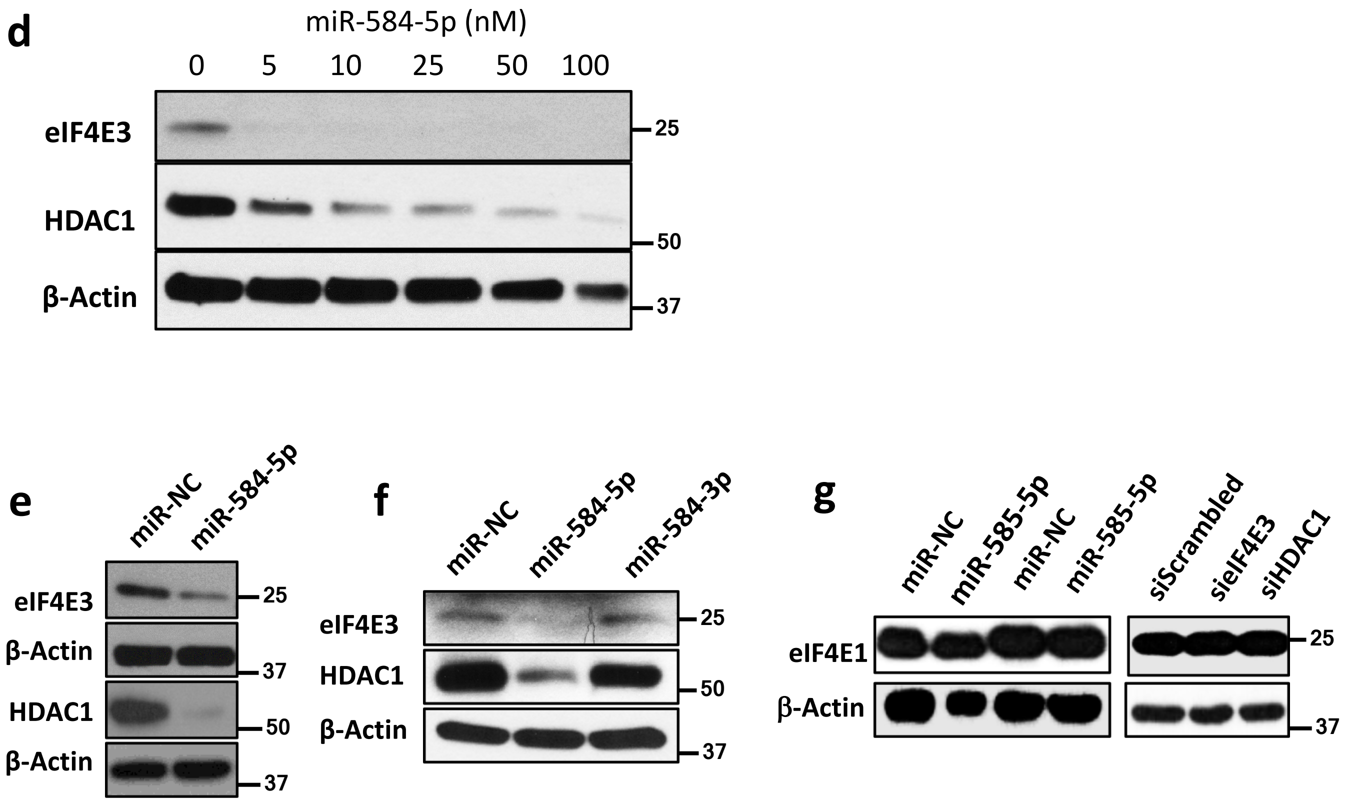

Supplementary Fig. 3. Analysis of enriched biological processes and target genes in *miR-584-5p* treated MB cells. (a) GSEA and IPA analyses showing altered biological processes in miR-584-5p mimic transfected MB cells (b) GSEA analysis showing enrichment plots for highly altered pathways in miR-NC comared to *miR-584-5p*. **(c)** Volcano-plot of gene expression changes in D-425 and D-458 med cells treated with miR-NC or *miR-584-5p* mimic for 48 hours. Red dots represent *miR-584-5p* target genes. (**d**) Western blot analysis of DAOY cells transfected with miR-NC or increasing concentration of miR-584-5p mimic using antibodies against HDAC1 or eIF4E3. β-actin was used as a loading control. Gel picture is representative of three independent experiments. **(e)** Western blot analysis of primary MB BT-50 cells transfected with miR-NC or miR-584-5p mimic using antibodies against HDAC1 or eIF4E3. β-actin was used as a loading control. Gel picture is representative of three independent experiments. **(f)** Western blot analysis of DAOY cells transfected with miR-NC, *miR-584-5p* or *miR-584-3p* using antibodies against HDAC1 or eIF4E3. β-actin was used as a loading control. Gel picture is representative of three independent experiments. **(g)** Western blot analysis of DAOY cells transfected with miR-NC, scrambled-siRNA, *miR-584-5p or* eIF4E3/HDAC1-siRNA using antibodies against eIF4E1. β-actin was used as a loading control. Gel picture is representative of three independent experiments.

Supplementary Fig 4

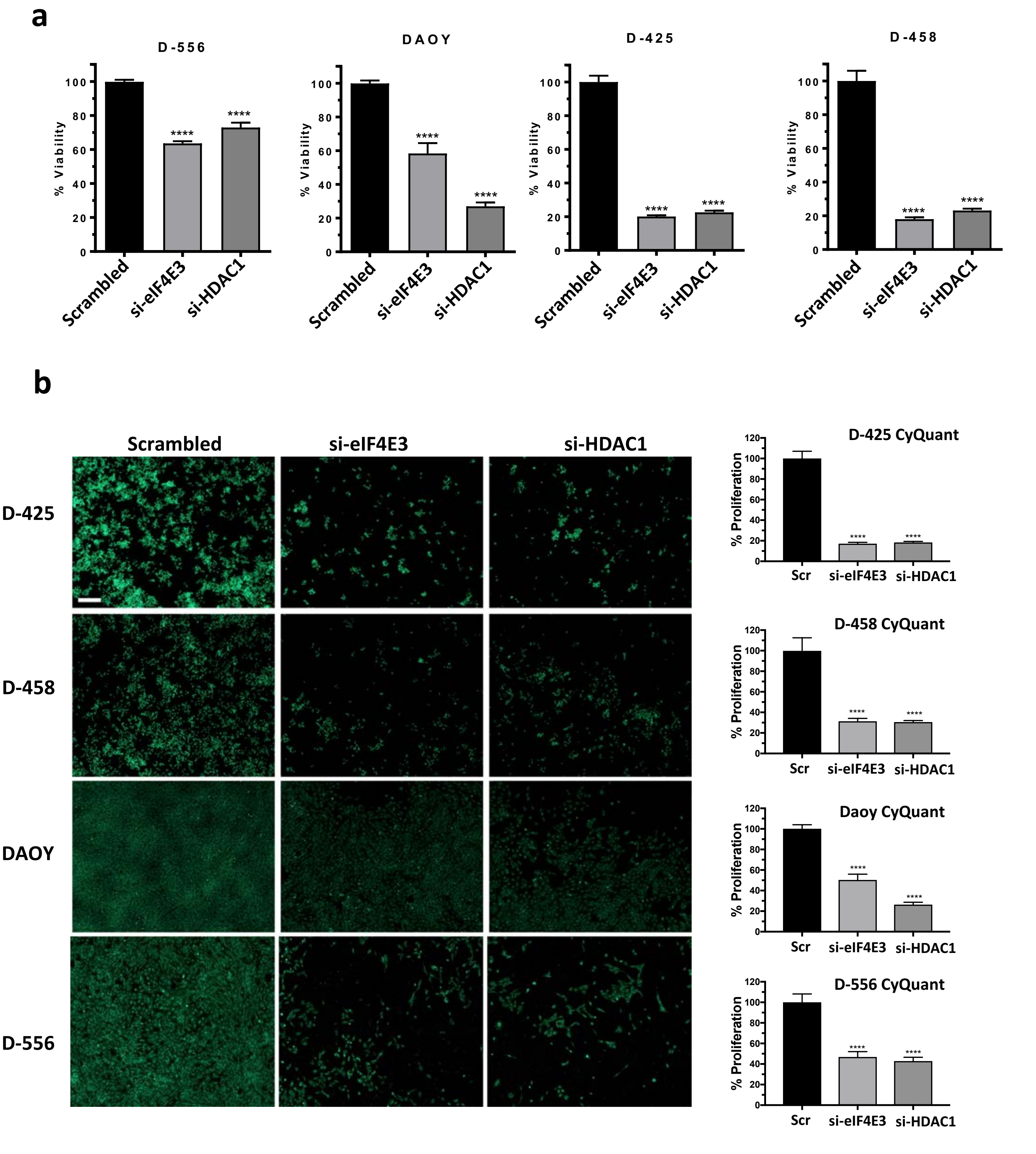

Supplementary Fig 4

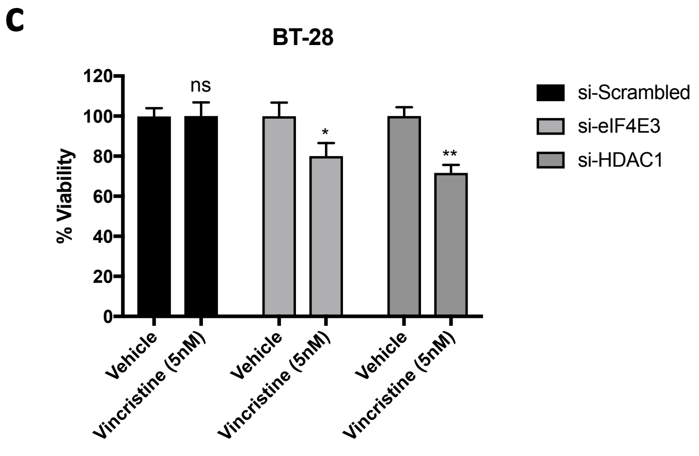

Supplementary Fig. 4. ***MiR-584-5p* target genes eIF4E3 and HDAC1 support MB growth. (a)** Bar graphs show viability of D556, D-425Med, D-458Med and DAOY cells transfected with 50 nM of scrambled, eIF4E3 or HDAC1 siRNAs. Cell viability was assessed using alamarBlue® cell viability assay. Error bars represent the mean ± SEM of three independent experiments. p-Value was calculated using one-way ANOVA followed by Dunnett's multiple comparisons test. **(b)** Representative images of D-556, D-425Med, D-458Med and DAOY cells transfected with 50 nM of scrambled, eIF4E3 or HDAC1 siRNAs and stained with CyQuant cell proliferation nuclear stain. Scale bar represents 50µM. Bar graphs show quantification of CyQuant fluorescence signal of of scrambled, eIF4E3 or HDAC1 siRNA transfected D556, D-425Med, D-458Med and DAOY cells. Error bars represent the mean ± SEM of three independent experiments. p-Value was calculated using one-way ANOVA followed by Dunnett's multiple comparisons test. **(c)** Bar graphs showing % viability of BT-28 cells transfected with scrambled, eIF4E3 or HDAC1 siRNA and treated with vehicle or VCR (vincristine). Error bars represent the mean ± SEM of three independent experiments.

Supplementary Fig 5

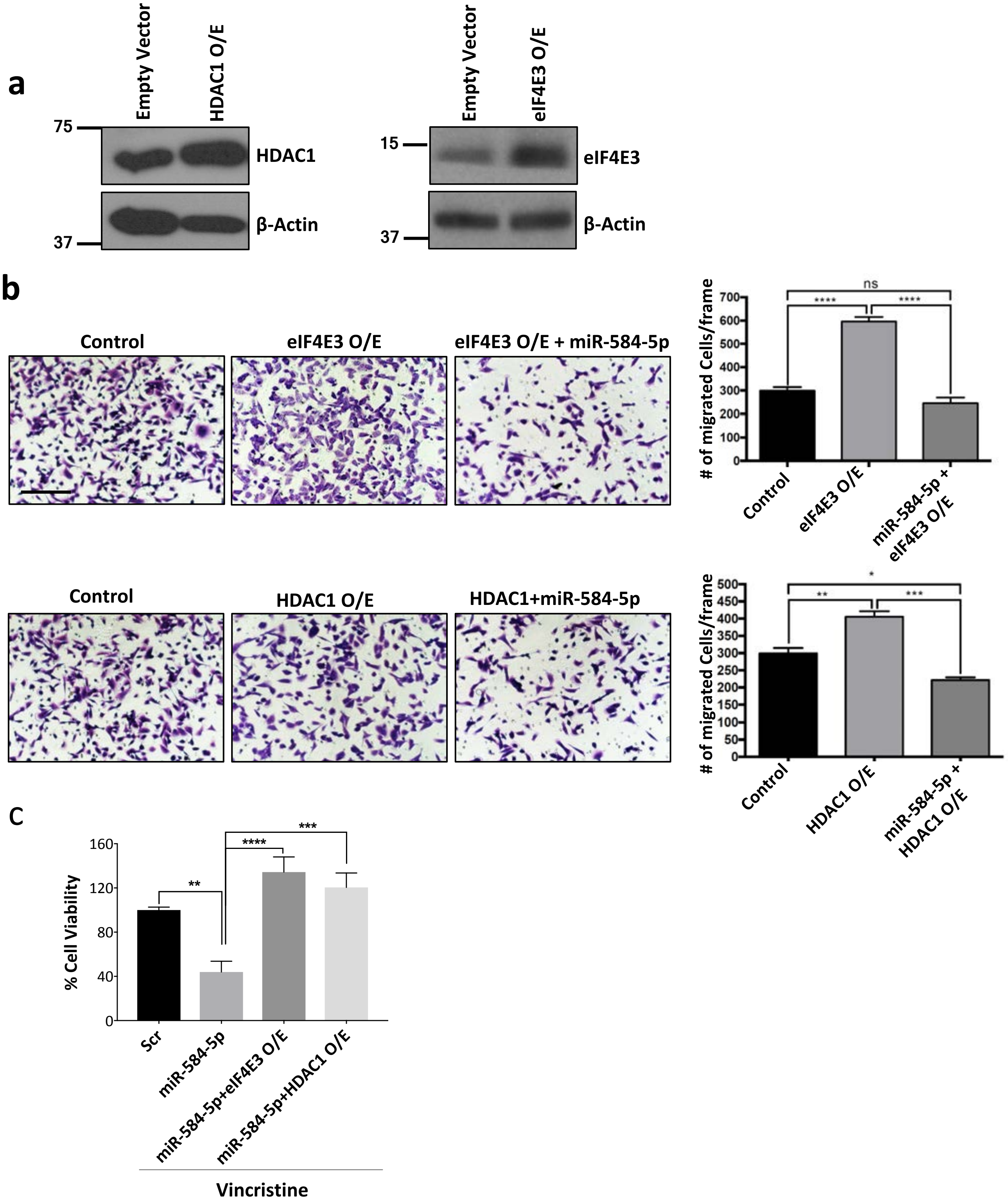

Supplementary Fig. 5. ***MiR-584-5p*** **rescues cancer cell migration promoting effects of eIF4E3 and HDAC1. (a)** Western blot analysis of D-556 cells transfected with empty vector, eIF4E3 or HDAC1 expression vector using antibodies against eIF4E3 and HDAC1. β-actin was used as a loading control. Gel picture is representative of three independent experiments. **(b)** Photomicrographs show migrated D-556 cells transfected with control plasmid or eIF4E3/HDAC1 expression vector or co-transfected with eIF4E3/HDAC1 expression vector and miR-584-5p mimic. Scale bar represents 50μM. Bar graph shows number of migrated cells in D-556 cells transfected with control plasmid or eIF4E3/ HDAC1 expression vector or co-transfected with eIF4E3/HDAC1 expression vector and miR-584-5p mimic counted microscopically. p-Value was calculated using one-way ANOVA followed by Sidak's multiple comparisons test. Error bars represent the mean ± SEM of three independent experiments. **(c)** Bar graphs show viability of DAOY cells co-transfected with scrambled (Scr), miR-584-5p, miR-584-5p + eIF4E3 or HDAC1 expression vectors and treated with vincristine (5nM). Cell viability was assessed using alamarBlue® cell viability assay. Error bars represent the mean ± SEM of three independent experiments. p-Value was calculated using one-way ANOVA followed by Dunnett's multiple comparisons test. ****$P < 0.0001$, *** $P < 0.001$, **$P < 0.01$, * $P < 0.05$.

Supplementary Fig 6

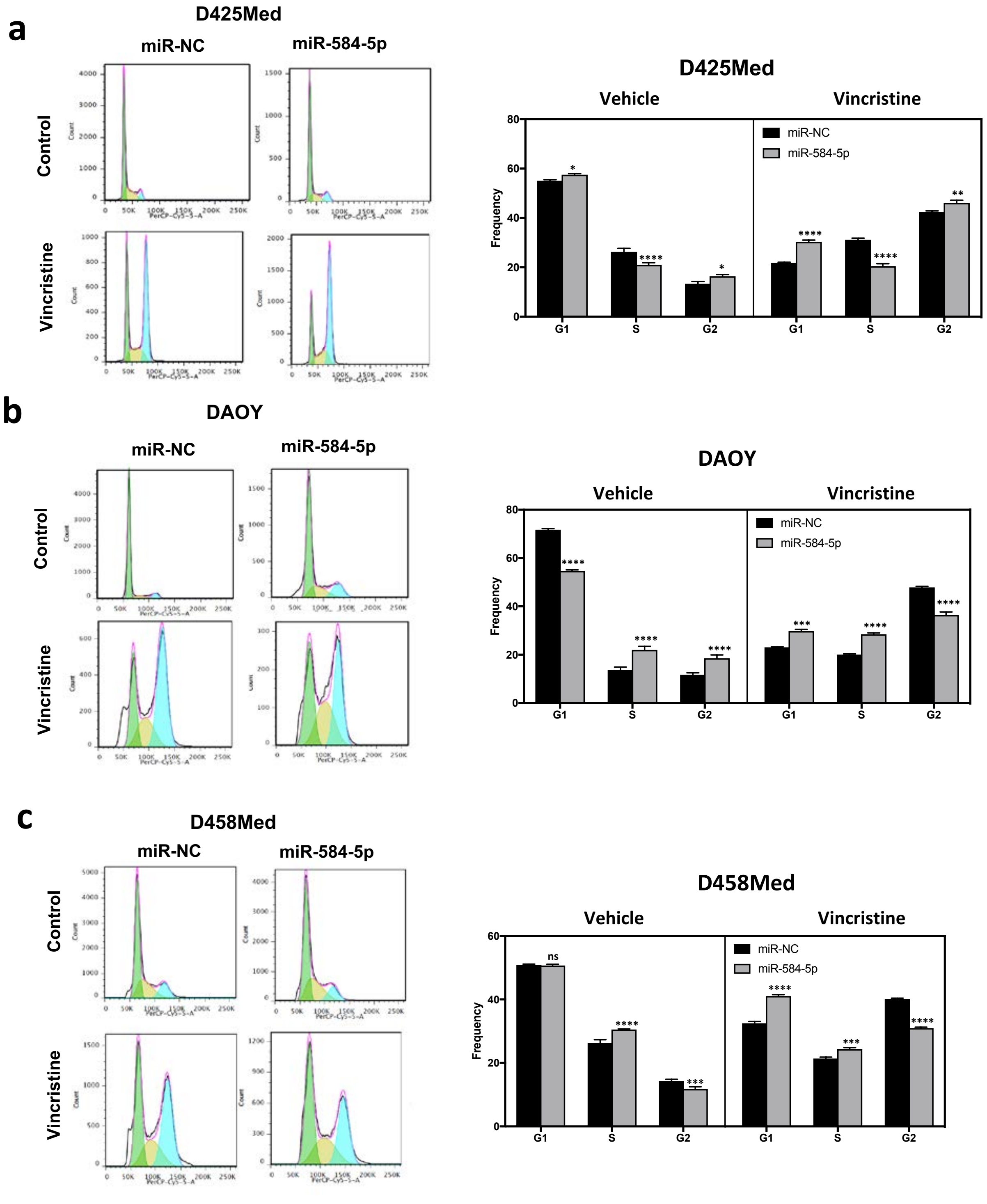

Supplementary Fig. 6. *MiR-584-5p* inhibits cell cycle progression in MB cells. (**a, b, c**) FACS analysis showing cell cycle progression in D-425Med (a), DAOY (b) and D-548Med (c) cells transfected with miR-NC or *miR-584-5p* and treated with vehicle or VCR. Bar graphs show DNA histograms quantified using the FlowJo software. P-Values were determined by two-way ANOVA followed by Tukey's multiple comparisons test. Error bars represent the mean ± SEM of three independent experiments.

Supplementary Fig 7

a

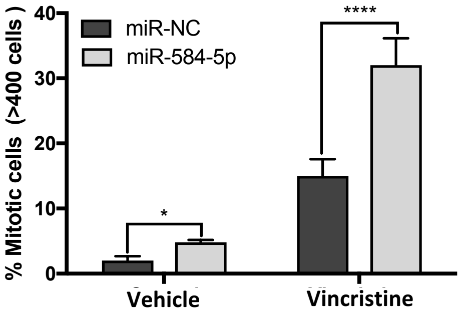

b

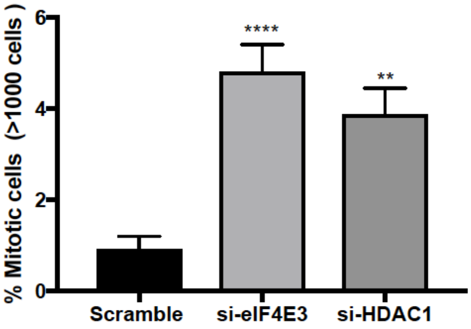

c

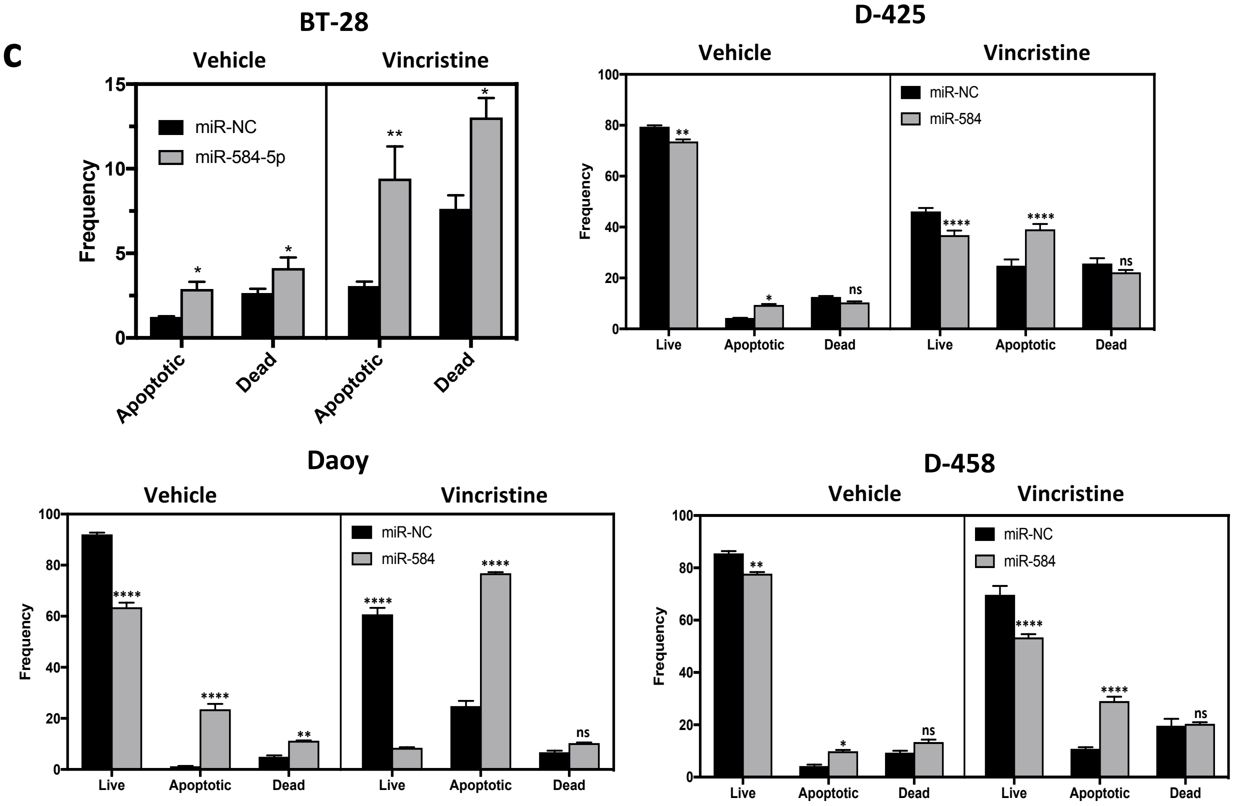

Supplementary Fig. 7. *MiR-584-5p* inhibits cell cycle progression and induces apoptosis in MB cells. (a) Bar graph showing analysis of Histone H3 pSer10 positive DAOY cells transfected with miR-NC or miR-584-5p and treated with vehicle or VCR. p-Values were calculated using standard student t-tests. Error bars represent the mean ± SEM of three independent experiments. **(b)** Bar graph showing analysis of Histone H3 pSer10 positive DAOY cells transfected with scrambled or eIF4E3/HDAC1-siRNA. Error bars represent the mean ± SEM of three independent experiments. ****P < 0.0001; **P<0.01. **(c)** FACS analysis of Annexin V-FITC positive cells in BT-28, D-425Med, D-458Med and DAOY cells transfected with miR-NC or *miR-584-5p* and treated with vehicle or VCR. Bar graphs show dot plots quantified using the FlowJo software. P-Values were determined by 2-way ANOVA followed by Tukey's multiple comparisons test. Error bars represent the mean ± SEM of three independent experiments. ****P < 0.0001, *** P < 0.001, **P < 0.01, * P < 0.05.

Supplementary Fig 8

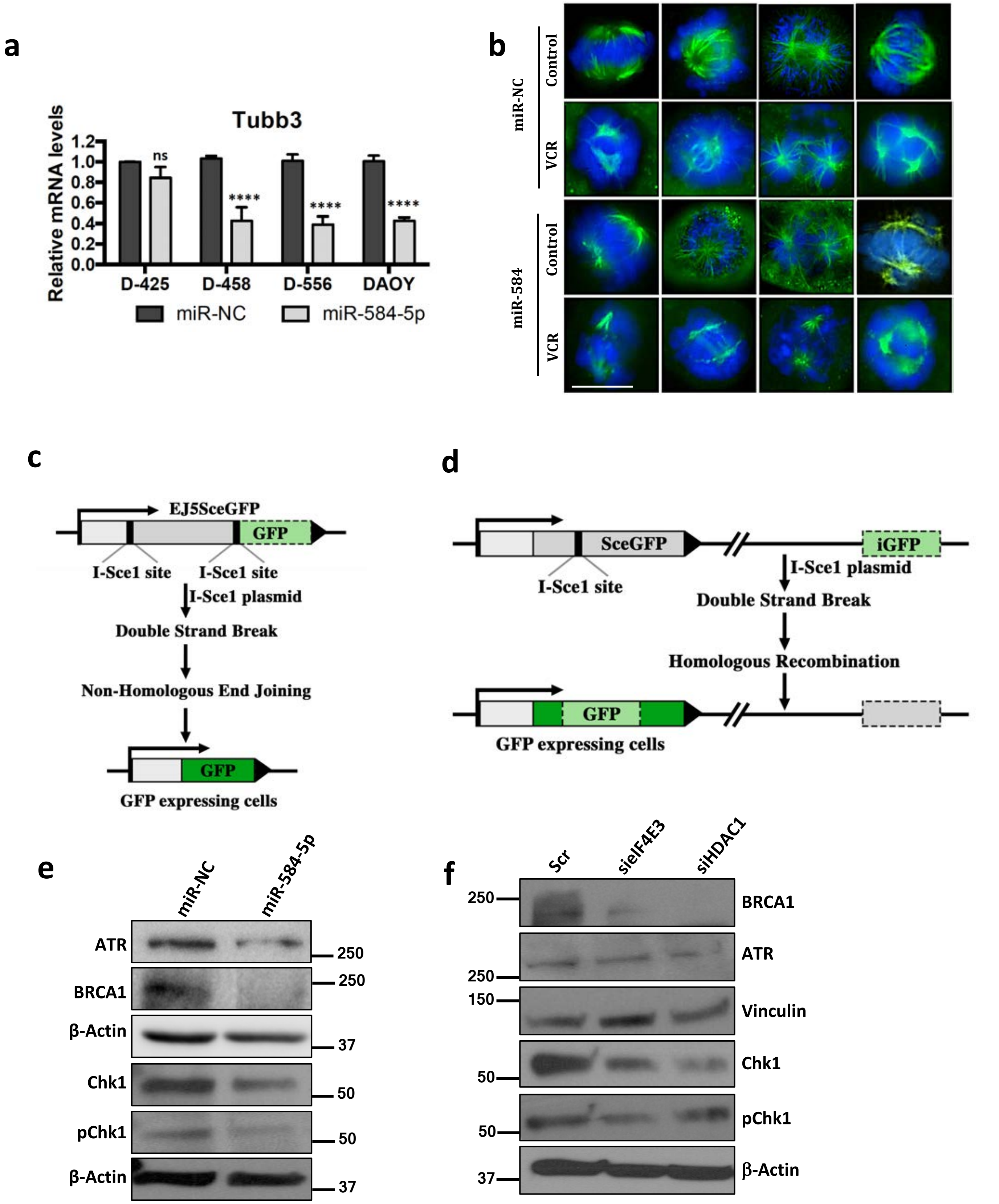

Supplementary Fig. 8. ***MiR-584-5p*** **mimic and eIF4E3/HDAC1 silencing induce spindle defects** (**a**) q-PCR analysis of TUBB3 in D-556, D-425Med, D-458Med and DAOY cells transfected with miR-NC or *miR-584-5p* mimic. P-Values were calculated using standard student t-tests. Error bars represent the mean ± SEM of three independent experiments (performed in triplicate for each experiment). (**b**) Representative immunofluorescence images of defective mitotic spindles in DAOY cells transfected with 50 nM of miR-NC or *miR-584-5p mimic*. DNA is stained with DAPI (blue), while spindle is stained with β-tubulin (green). Scale bar represents 10μM. **(c, d)** Schematics of the EJ5SceGFP (c) and DR-GF (d) reporter assay that measures NHEJ and HR, respectively. **(e, f)** Western blot analysis of D-556 cells treated with miR-NC or miR-584-5p mimic (e) or scrambled or eIF4E3/HDAC1-siRNA (f) using antibodies against indicated proteins. Gel pictures are representative of three independent experiments. Vinculin and β-actin were used as loading controls.

Supplementary Figure 9

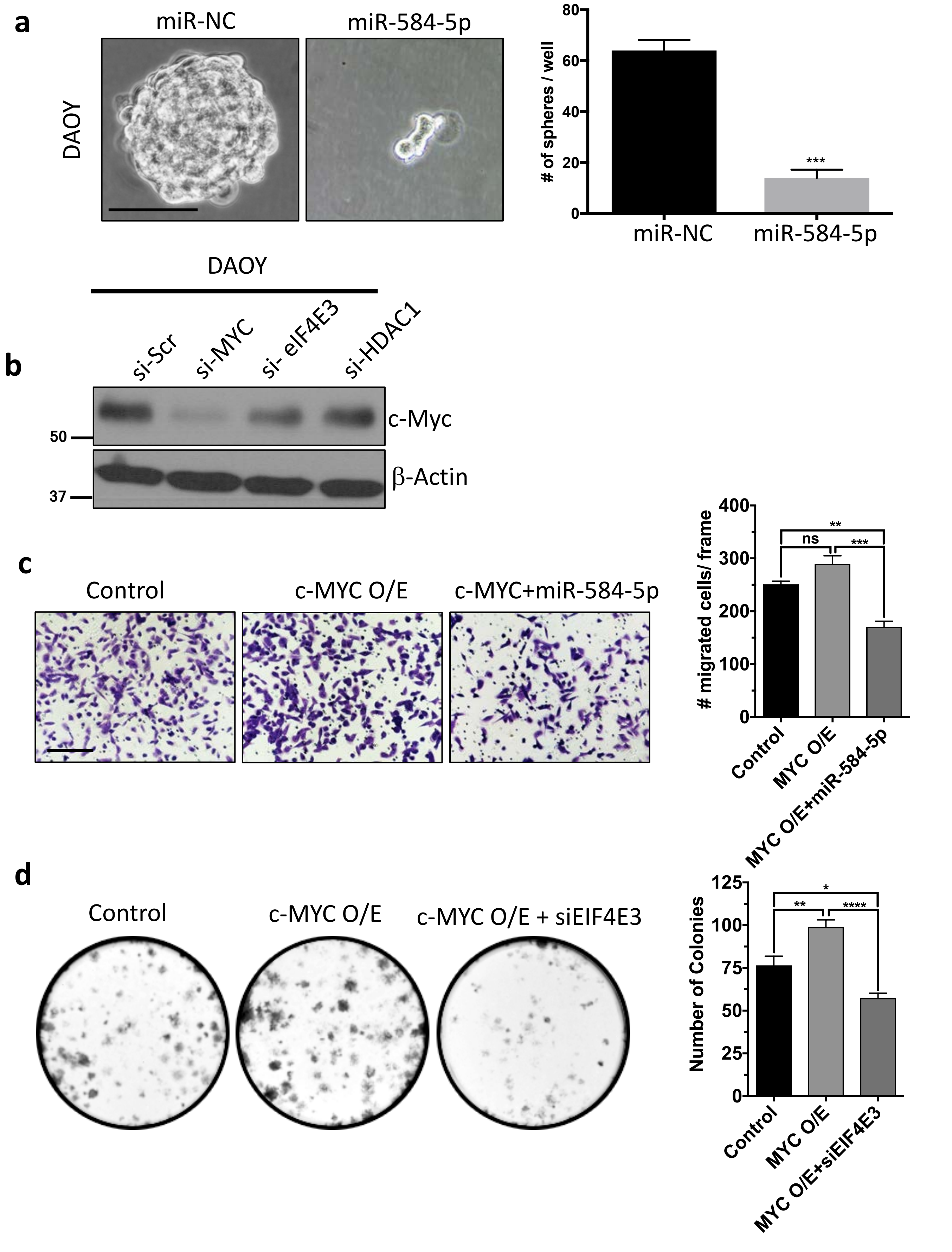

Supplementary Fig. 9. *MiR-584-5p*-eIF4E3 regulates c-Myc expression and MB stem cell self-renewal. (**a**) Medullospheres grown from DAOY cells transfected with miR-NC or *miR-584-5p* mimic. Scale bar represents 50µM. Bar graphs show number and size of medullospheres obtained from DAOY cells transfected with miR-NC or *miR-584-5p* mimic. The *p*-value was calculated using a standard Student *t*-test. Error bars represent mean ± SEM of three independent experiments (performed in triplicate for each experiment). **(b)** Western blot analysis of DAOY cells transfected with scrambled, eIF4E3- or HDAC1-siRNA using antibody against c-myc. β-actin was used as a loading control. Gel picture is representative of three independent experiments. (**c**) *miR-584-5p* rescues cancer growth promoting effects of MYC. Photomicrograph showing migrated DAOY cells transfected with c-MYC expression vctor or c-MYC + *miR-584-5p* mimic. Scale bar represents 50µM. Bar graphs show number of migrated cells in DAOY cells transfected with c-MYC expression vctor or c-MYC + *miR-584-5p* mimic counted microscopically in ten different fields. Error bars show mean ± SEM of three independent experiments. (**d**) eIF4E3 depletion rescues cancer growth promoting effects of c-MYC. Clonogenic assay of D556 cells transfected with empty vector, c-Myc expression vector or c-Myc expression vector + eIF4E3-siRNA. Bar graph shows crystal violet–stained colonies counted microscopically.

Supplementary Figure 10

a

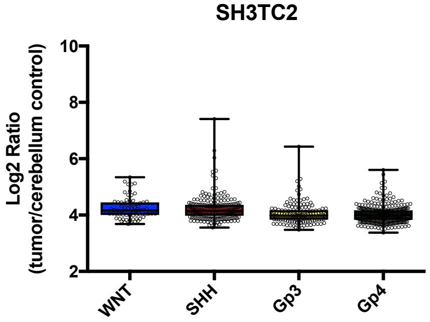

b

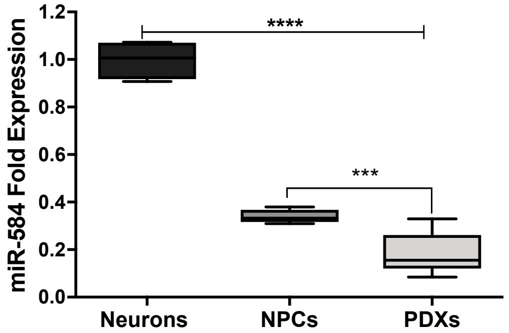

Supplementary Fig. 10. *MiR-584-5p* and its host gene SH3TC2 are expressed at lower levels in MB. (**a**) Meta-analysis of GSE85217 data set showing SH3TC2 expression levels in human medulloblastoma samples (WNT: n=763; SHH: n=223; Gp3: n=144; Gp4: n=326). (**b**) *Taq*man qPCR analysis of miR-584-5p expression in iPSC-derived mature neurons, neural progenitor cells (NPCs), and MB patient derived xenografts (PDXs; *n* = 4). The *p*-values were calculated using one-way ANOVA followed by Sidak's multiple-comparisons test. Error bars represent mean ± SEM of three independent experiments. Whiskers in the box plots represent minimum to maximum value. The center line in the box represents the median value. Black open circles in the box (a) represent individual MB patient tumor sample.

Supplementary Figure 11

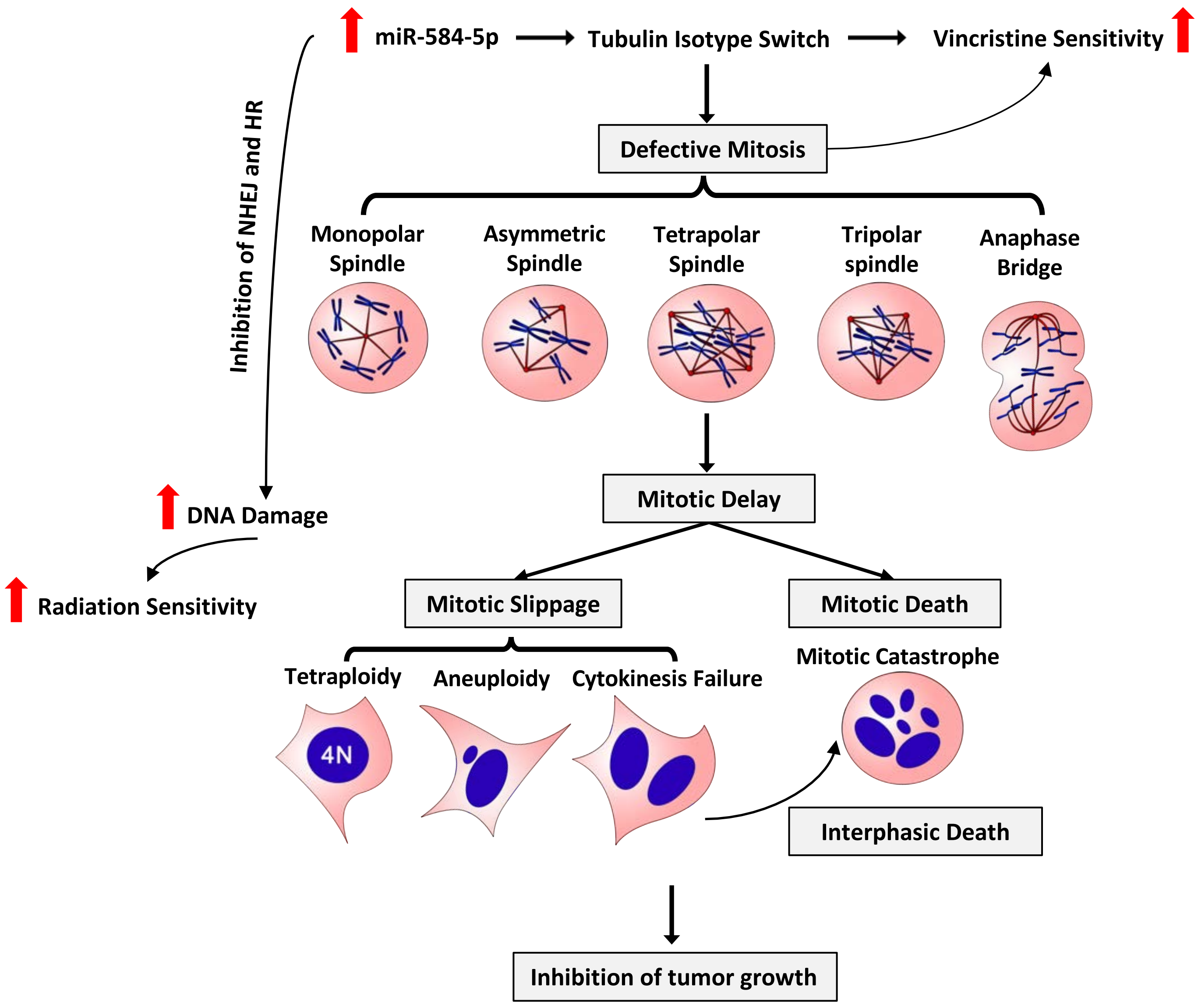

Supplementary Fig. 11. Model showing *miR-584-5p* mechanisms of action. We show that miR-584-5p induces mitotic death in MB cells. Furthermore, we show that miR-584-5p induces DNA damage and blocks DNA repair events. We propose that *miR-584-5p* mimic–treated cells with unrepaired DNA damage enter mitosis where they incur *miR-584-5p*–induced spindle defects, resulting in mitotic catastrophe and death of MB cells. In addition, we propose that miR-584-5p-induced mitotic slippage causes aneuploidy. Since aneuploidy can generate double-stranded breaks it is likely that that improper repair of these breaks due to miR-584-5p's ability to block NHEJ/HR results in MB growth inhibition and sensitization of IR response in MB cells. Alteration in tubulin isoform expression is known to confer VCR resistance in cancer cells. Furthermore, tubulin aggregation and consequently inhibition of microtubule assembly and mitotic disruption are mechanisms by which VCR inhibits cancer cell growth. Therefore, we posit that miR-584-5p potentiates VCR response by affecting tubulin expression and microtubule dynamics in MB cells.

Supplementary Figure 12

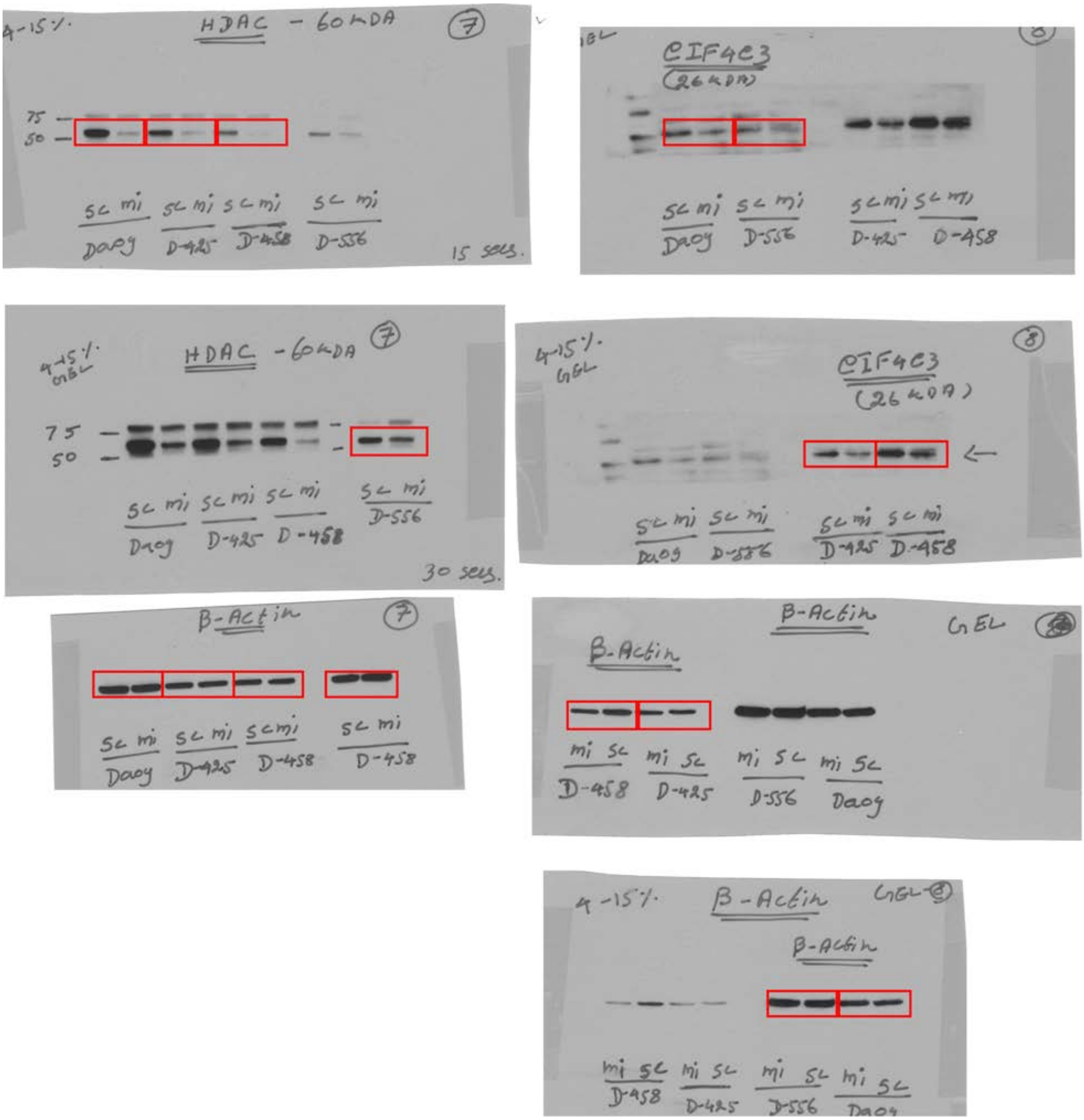

Uncropped Films for Figure 4d

Supplementary Figure 12

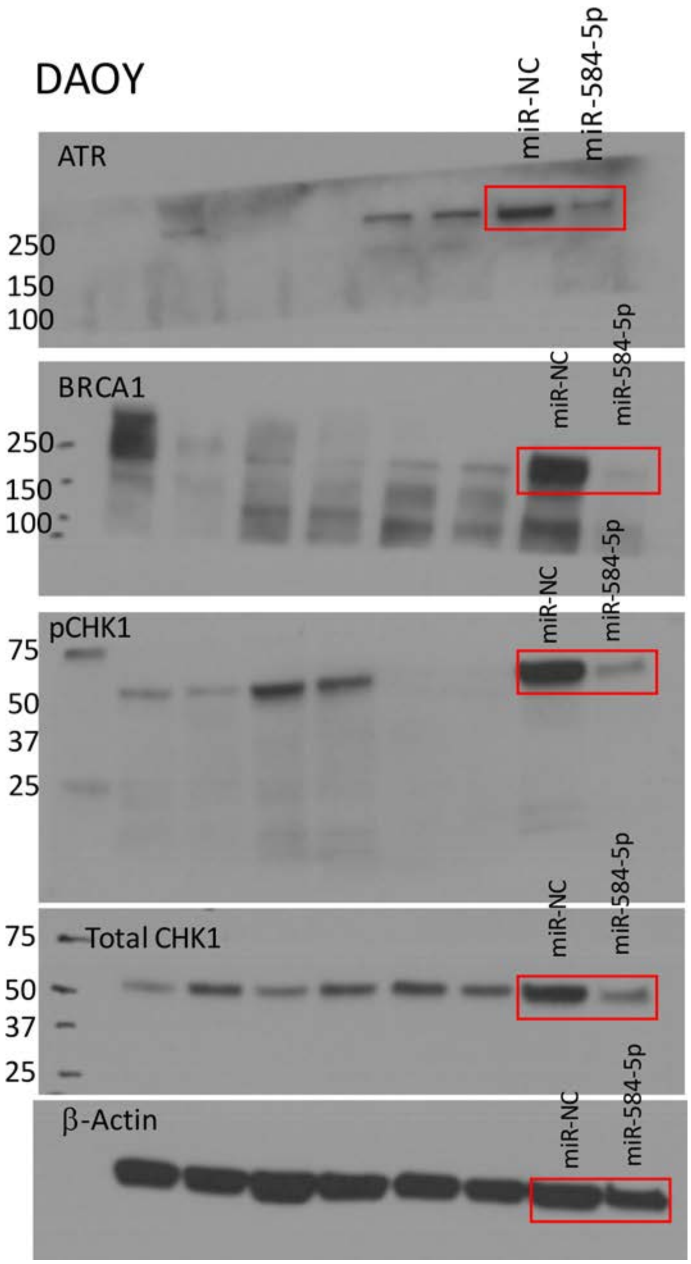

Uncropped Films for Figure 9a

Supplementary Figure 12

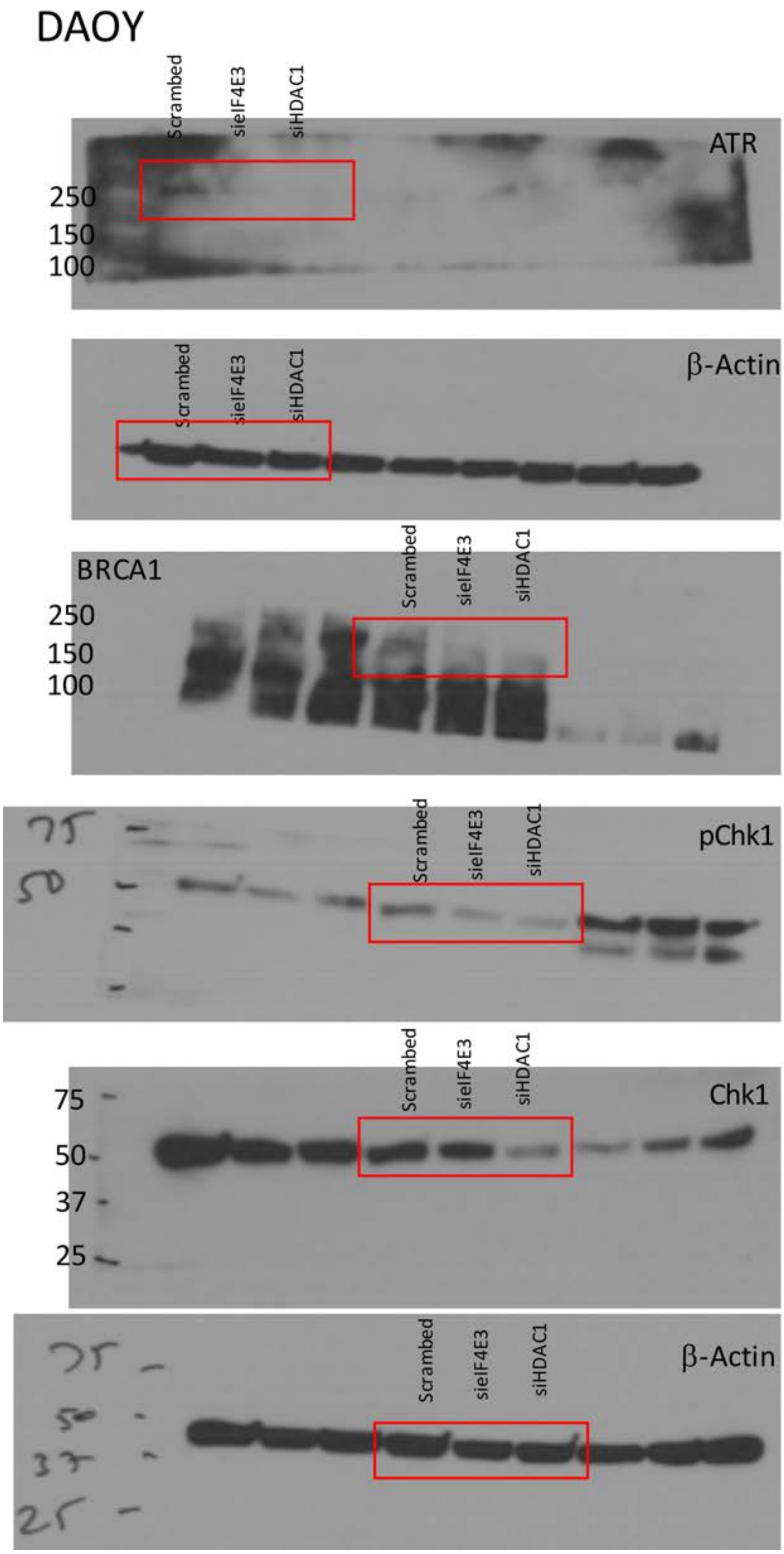

Uncropped Films for Figure 9b

Supplementary Figure 12

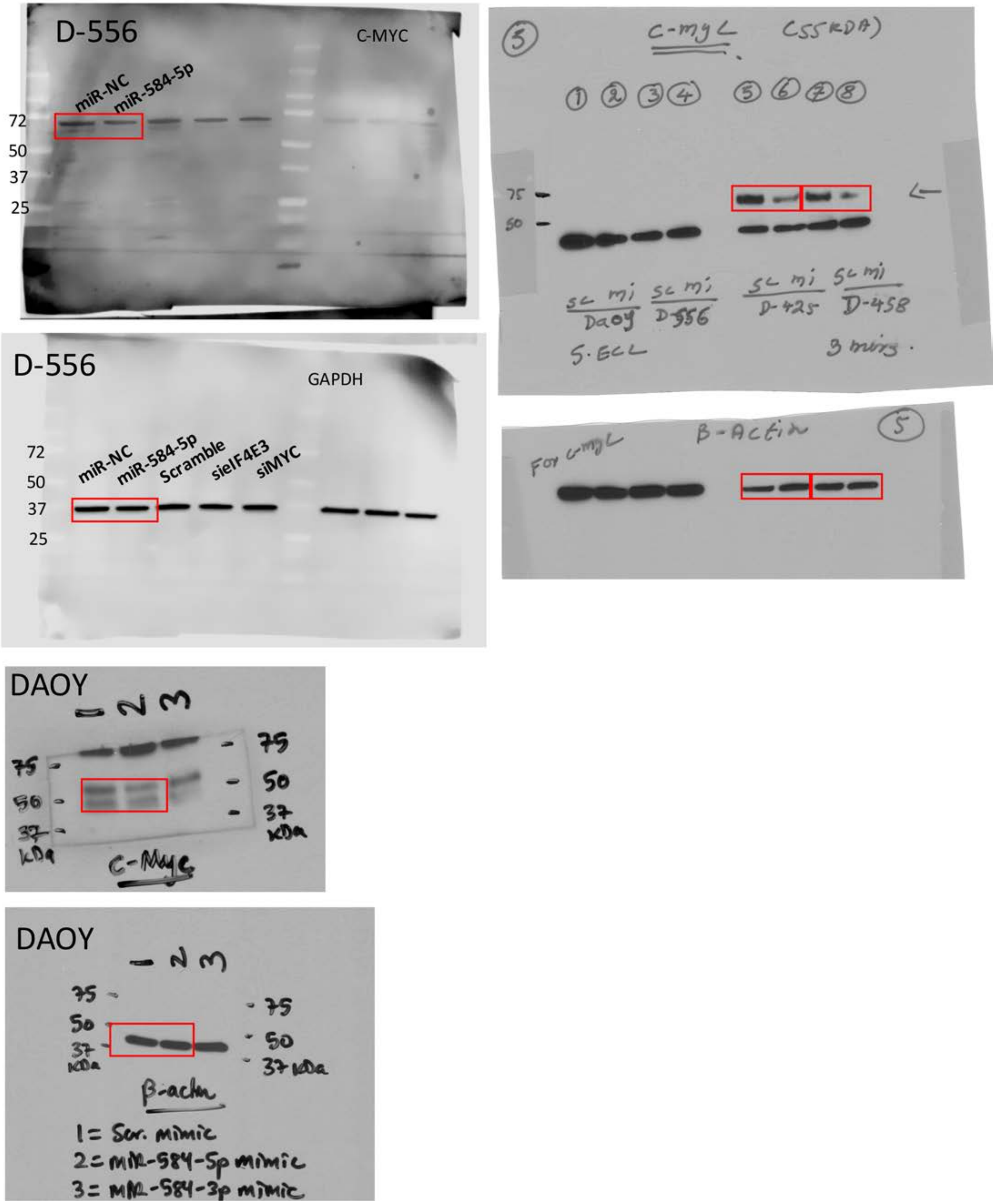

Uncropped Films for Figure 9g

Supplementary Figure 12

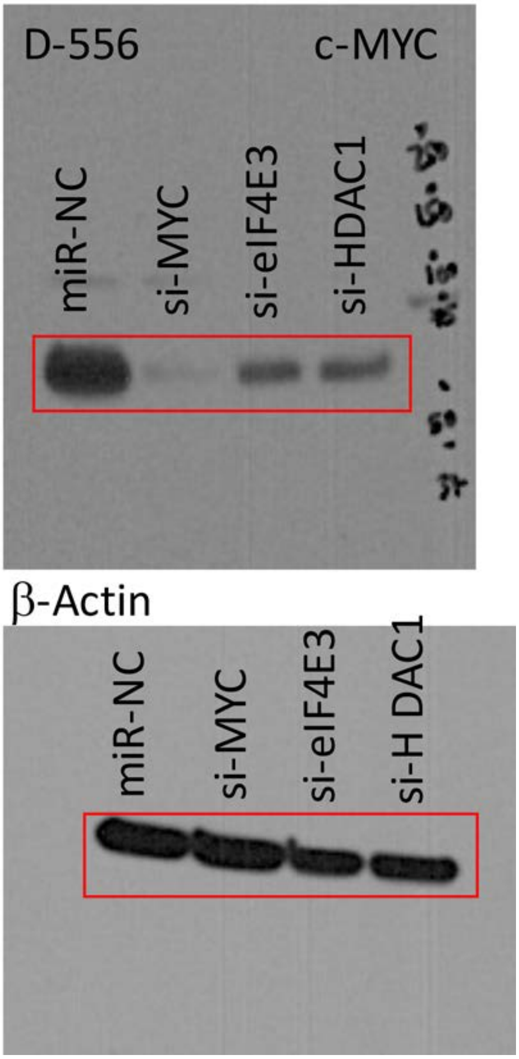

Uncropped Films for Figure 9h

Supplementary Figure 12. Uncropped western blots. Cropped sections used as figures in the manuscript are marked as a red box.
