# Supplementary material for: MiR-584-5p potentiates vincristine and radiation response by inducing spindle defects and DNA damage in medulloblastoma
Source: Nat Commun. 2018 Oct 31;9:4541. doi: 10.1038/s41467-018-06808-8 (PMC6208371; doi:10.1038/s41467-018-06808-8)
Supplement: Supplementary file 2 — Description of Additional Supplementary Files [file 41467_2018_6808_MOESM2_ESM.docx]

**Title:** Supplementary Dataset 1

**Description:** List of common differentially expressed genes in D425Med and D458Med medulloblastoma cells overexpressing miR-584-5p compared to scrambled.
